# Delineating specific regions of N- terminal domain of T3SS ATPase YsaN of *Yersinia enterocolitica* governing its different oligomerization states

**DOI:** 10.3389/fmolb.2022.967974

**Published:** 2022-09-08

**Authors:** Rajeev Kumar, Chittran Roy, Saumen Datta

**Affiliations:** Structural Biology and Bioinformatics Division, Council of Scientific and Industrial Research–Indian Institute of Chemical Biology, Kolkata, West Bengal, India

**Keywords:** T3SS, ATPases, hexamer, dodecamer, negative-TEM

## Abstract

Oligomerization of YsaN, a putative T3SS-ATPase is a necessary and crucial event for T3SS functioning in *Y. enterocolitica*. Different oligomeric states have been proposed for similar ATPases, yet, the true nature of its activation and formation of different oligomers is still poorly understood. *In-vitro* studies of YsaN reveal that its activation and oligomerization depend on its N-terminal region and occur as a result of active catalysis of ATP in an ATP concentration-dependent manner following two-step cooperative kinetics. Also, the N-terminal 83 amino acid residues of YsaN are crucial for higher-order oligomer formation while YsaN∆83 is capable of hexamer formation upon oligomerization. Enzyme kinetics study shows reduced ATPase activity of YsaN∆83 (3.19 ± 0.09 μmol/min/mg) in comparison to YsaN (9.076 ± 0.72 μmol/min/mg). Negative-TEM study of YsaN and YsaN∆83 oligomer suggests that the formation of higher-order oligomer (probably dodecamer) occurs by stacking of two hexamers through their N-terminal faces involving N-terminal 83 amino acid residues which have been further supported by the docking of two hexamers during the *in-silico* study. These results suggest that YsaN is an oligomerization-activated T3SS ATPase, where distinct regions of its N-terminal domain regulate its different oligomeric nature and is essential for its activation.

## 1 Introduction

The type three secretion system (T3SS) has been regarded as major virulence determining factor in numerous plants and animal pathogenic bacteria (Coburn et al., 2007; Buttner, 2012). A broad spectrum of diseases is caused by pathogenic bacteria such as enteric infections caused by enteropathogenic *E. coli*, *Shigella*, *Salmonella,* and *Y. enterocolitica* containing T3SS*.* These pathogens use T3SS to inject effector toxins directly inside the host cell to manipulate host cellular processes. Mutations, deletions, or blocking of the T3SS apparatus components result in reduced virulence and disease manifestations in mouse model experiments (Coburn et al., 2007). T3SS has been predicted to be evolved from flagellar T3SS (fT3SS) through horizontal gene transfer and both of them share a common ancestor. Both T3SS and fT3SS share many structurally similar components at their core (Gophna et al., 2003; Diepold and Armitage, 2015). Bacterial T3SS is a complex structure composed of approximately 20 different proteins and broadly can be divided into three basic parts namely the basal body, needle complex, and a large cytosolic component known as the sorting platform or the C- ring complex. The basal body consists of two co-axial homomeric protein complex rings across the inner and outer plasma membrane including the peptidoglycan layer. The needle is present in association with the outer ring projecting away from the bacterial membrane providing approximately 1.5–2.5 nm conduit for translocation of unfolded effectors. Tip complex protein associated with the needle helps the bacteria to integrate with the host plasma membrane. Once the association with the host membrane is established, the export apparatus helps in the translocation of effectors through the needle to the host cell cytoplasm (Kubori et al., 1998; Ghosh, 2004; Diepold and Wagner, 2014). Previous structural studies involving Cryo-Electron microscopy have revealed the global structure of T3SS Injectisome. However, the precise details of the cytoplasmic components are largely undetermined because of their dynamic nature of association with the injectisome.

Energy for unfolding and translocation of effectors is provided by both, Proton Motive Force (PMF) (Wilharm et al., 2004) and ATP hydrolysis by a highly conserved ATPase ring complex which remains in close association with the active needle complex at the sorting platform (Lee and Rietsch, 2015). These ATPases also have structural and functional similarities with the F_0_- F_1_ ATPase—an AAA + ATPase (ATPase associated with various cellular activities) (Akeda and Galan, 2005; Yoshida et al., 2014). Structural and functional similarity between T3SS ATPases and the AAA + enzymes suggest the possibility of a universal model of mechanism among such molecular motors.

Based on the results from Electron microscopy, high-resolution Cryo-electron tomography, and biochemical studies, it has already been suggested that T3SS ATPases function as homo-oligomers namely hexamer (Hu et al., 2017; Halder et al., 2019) and dodecamer (Pozidis et al., 2003; Muller et al., 2006) which are located at the central pore of the T3SS apparatus on the cytoplasmic side. Besides, previous studies have also shown that during effector unfolding and translocation through the needle complex, the effector chaperone complex docks to the C- terminal side of the ATPase ring complex at the export gate. Also, the C-terminal side of this ATPase ring complex faces the pore of the needle complex (Akeda and Galan, 2005; Allison et al., 2014; Majewski et al., 2019).

*Y. enterocolitica* uses contact-dependent T3SS (Abrusci et al., 2013) for the delivery of anti-host effector proteins directly into the eukaryotic host cell (Cornelis, 2002; Edgren et al., 2012; Bent et al., 2013). In addition to fT3SS, *Y. enterocolitica* maintains two distinct, independently regulated T3SSs. The first one is *Ysa- Ysp* T3SS encoded by *Ysa* Pathogenicity Islands located on the chromosome and another one is *Ysc- Yop* T3SS which is encoded by ≈70 Kb virulent plasmid pYV/pCD1 (Cornelis et al., 1998; Foultier et al., 2002). The *Ysa- Ysp* T3SS is required for the gastrointestinal phase of infection and intracellular survival of *Y. enterocolitica* within macrophages (Bent et al., 2015) and is important in virulence (Haller et al., 2000). In our previous study, it was shown that YsaN, a T3SS ATPase in *Y. enterocolitica* encoded by *Ysa- Ysp* T3SS is a magnesium-dependent ATPase and the probable dodecamer state is the most active form of YsaN. Moreover, it was also shown that the N- terminal 1–20 amino acid residues of YsaN is crucial for YsaL binding (a negative regulator of YsaN ATPase) (Chatterjee et al., 2013).

In the present study, we have performed the *in-vitro* characterization of untagged YsaN and various deletion constructs. Another focus of this study is the characterization of the formation of different functionally relevant higher-order oligomers based on its N- terminal region i.e., partially active hexamer and the most active higher-order oligomer forms of YsaN. The present study indicates the involvement of distinct regions on the N- terminal domain of YsaN in the formation of hexamer and its higher-order oligomer. It has also been shown that the transition to a higher oligomer state of YsaN is an ATP concentration-dependent event following two-step kinetics. The existence of the hexamer as well as the higher-order oligomer complex has also been observed in negative TEM which suggest that the formation of higher-order oligomer occurs by stacking of two homo- hexamers through their N- terminal faces which are also supported by perfect docking between two hexamer rings during *in-silico* studies.

## 2 Materials and methods

### 2.1 Cloning expression and purification

**FIGURE 1:**
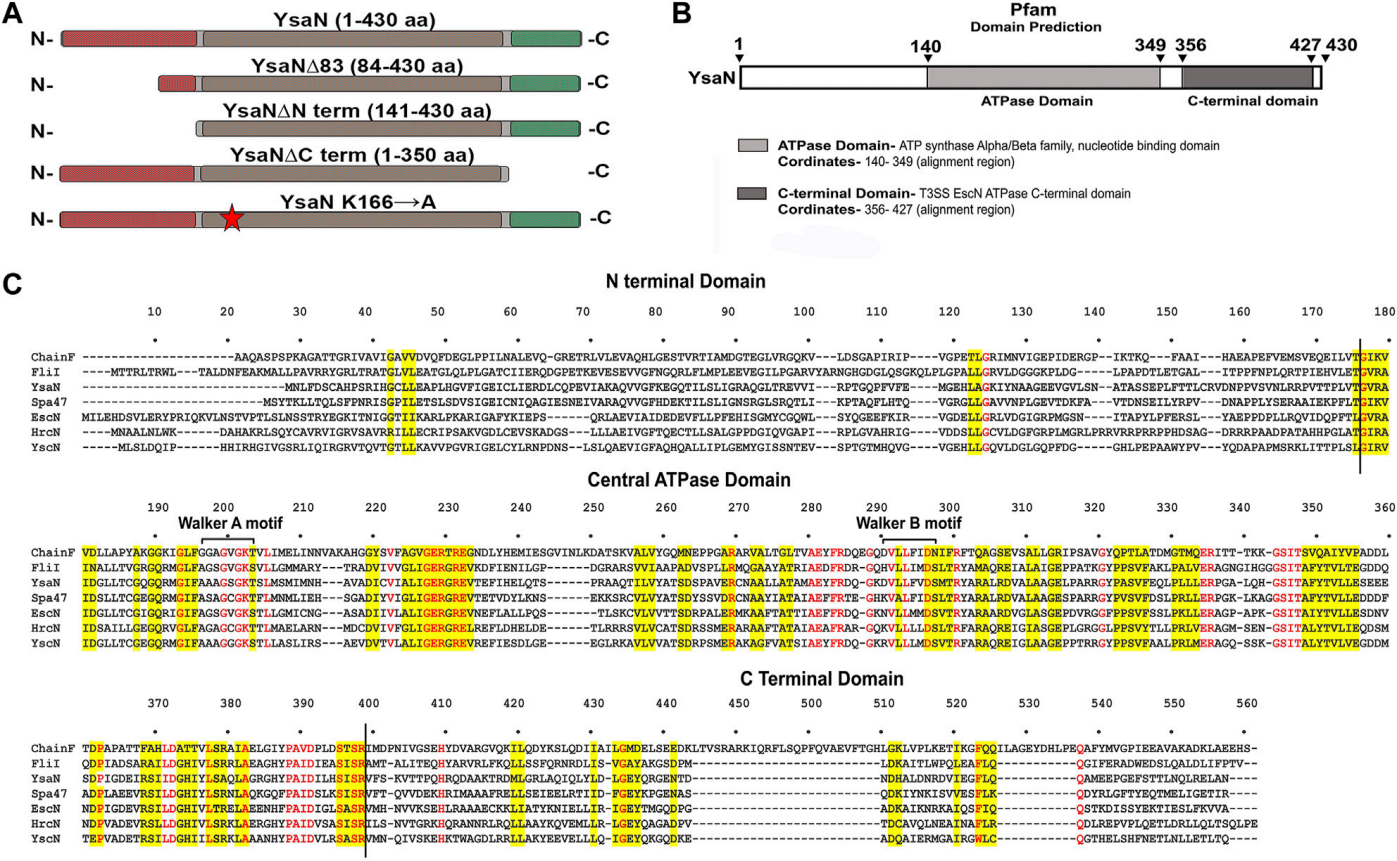
Bioinformatic analysis of YsaN- **(A)**, Linear representation of the YsaN constructs used in this study (based on domain analysis of YsaN using Pfam, also refer to Table 1 for complete details) Different color shading represents respective domains of YsaN, Red- represents N terminal domain, Gray- represents a central domain, and green- represents C-terminal domain. The red star shows site-directed mutation site on the central domain. **(B)**, Pfam Domain analysis of YsaN sequence. **(C)**, Multiple sequence alignment of YsaN and homologs (also refer to Supplementary Tables S2–S4 for details). The vertical black line represents the domain partition (i.e., N-terminal and C-terminal domains) of YsaN sequence based on Pfam domain analysis.

Plasmid vectors and constructs used in this study are given in Table 1. Genomic DNA was isolated from *Y. enterocolitica* 8081 culture according to (Wilson, 2001). *YsaN* wild-type gene was PCR amplified from this purified genomic DNA using *Pfu* DNA polymerase Thermo Scientific™ and cloned into *pET*22b∆50CPD vector containing C- terminal His- tagged cysteine protease domain (hereafter CPD vector) with Nde1 and BamH1 restriction sites. Similarly, other *YsaN* deletions (based on P*fam* domain analysis, refer to Figure 1B) were cloned in fusion with the CPD domain in the *pET*22b∆50CPD vector. Primers used in this study are listed in Supplementary Table S1. Cloning in CPD vector resulted in soluble expression of all YsaN deletions which were usually not soluble when expressed alone (data not shown) (Shen et al., 2009). DH5α was used as a cloning host in all cases. For recombinant expression of the protein, the plasmid constructs were transformed into BL21 DE3 chemically competent cells. IPTG (at 0.5 mM working concentration) was added after OD 600 reached 0.6 and kept for constant shaking for approximately 12–14 h at 298 K. Induced cells were harvested at 5,000 g at 277 K for 10 min. Cells were lysed in sonication buffer (50 mM Tris pH 8.0, 100 mM NaCl, 5% glycerol, and 5 mM imidazole) by ultrasonication method using a sonicator (Q-Sonica 125). Protease inhibitor PMSF at 1 mM working concentration was added to the resuspended pellet just before sonication. Centrifugation was done to remove the cellular debris at 18,000 g at 277 K for approximately 60 min. Protein was purified according to the CPD purification protocol (Shen et al., 2009) with few modifications. All the purification steps were carried out at 277 K in a cold room. Briefly, the clear supernatant was loaded onto a pre-equilibrated gravity-flow purification column, containing Nickel beads (Nickel Sepharose™ Fast Flow, GE Healthcare) followed by incubation of approximately 60 min. The beads were washed with two column volumes (CV) of wash buffer (50 mM Tris pH 8.0, 100 mM NaCl, 5% glycerol, and 35 mM imidazole). Then, the beads were mixed and incubated with CPD buffer (50 mM Tris pH 8.0, 100 mM NaCl, 5% glycerol) added with 100 µM (working) Inositol-6- phosphate (Phytic acid sodium salt hydrate, sigma) for approximately 60 min for CPD-His-tag removal. The incubated sample (containing untagged YsaN and other deletion proteins) was collected in a fresh collection tube and immediately injected into the gel filtration system AKTA prime plus (GE Healthcare). CPD buffer incubation time for more than 60 min was strictly avoided in our case to reduce the degradation of YsaN and other YsaN deletion proteins by CPD itself. Gel filtration was done to further purify the protein using the Hi-load Superdex 200 16/60 gel filtration column (GE Healthcare). Immediate gel filtration was necessary in this case to avoid further degradation of target proteins due to leached CPD from the nickel beads which was separated only after gel filtration. 250 mM Imidazole in elution buffer was used to elute the residual proteins bound to the beads. The corresponding peak in size exclusion respective to YsaN and its deletions were collected and analyzed by SDS gel electrophoresis. This purified protein sample was used for all downstream experiments. Freshly purified samples were used in all experiments each time.

**TABLE 1:** List of clones/vectors used in this study.

Vectors/Plasmid construct name	Details	Protein size (approximate molecular weight)	Reference(s)/source
pET22b∆50CPD^a^	*Vibrio cholerae* MARTX toxin cysteine protease domain with C terminal His Tag in pET22b	23.1 kDa	Gifted by Matthew Bogyo, Department of Pathology, Stanford School of Medicine, Stanford, California
YsaN	YsaN wild type full length (430 amino acids cloned in pET22b∆50CPD vector)	47.87 kDa	This Study
YsaN∆83	N terminal 1–83 amino acid deletion of YsaN (347 amino acids cloned in pET22b∆50CPD vector)	38.6 kDa	This Study
YsaN∆C-term	C terminal 357–430 amino acid deletion of YsaN (349 amino acids cloned in pET22b∆50CPD vector)	39.2 kDa	This Study
YsaN∆N-term	N terminal 1–140 amino acid deletion of YsaN (290 amino acids cloned in pET22b∆50CPD vector)	32 kDa	This Study
YsaN K166→ A	The lysine at the 166th position is mutated to alanine. It is a non-functional mutant of YsaN. (430 amino acid cloned in pET22b vector with C-terminal his tag.)	49 kDa	This study

a ∆ represents deletion in all cases.

### 2.2 Site-directed mutation

To create a non-functional Walker-A lysine to alanine mutant of YsaN i.e., YsaN K166→A, site-directed mutation was performed according to (Ho et al., 1989) in YsaN pET22b∆50CPD construct. Primers used for mutation studies are mentioned in Supplementary Table S1. The positive clones were verified by sequencing. The positive clone plasmid was further transformed into BL21 for further purification. ATPase assay was performed to verify its loss of activity (Supplementary Figure S5B).

### 2.3 ATPase assay

**FIGURE 2:**
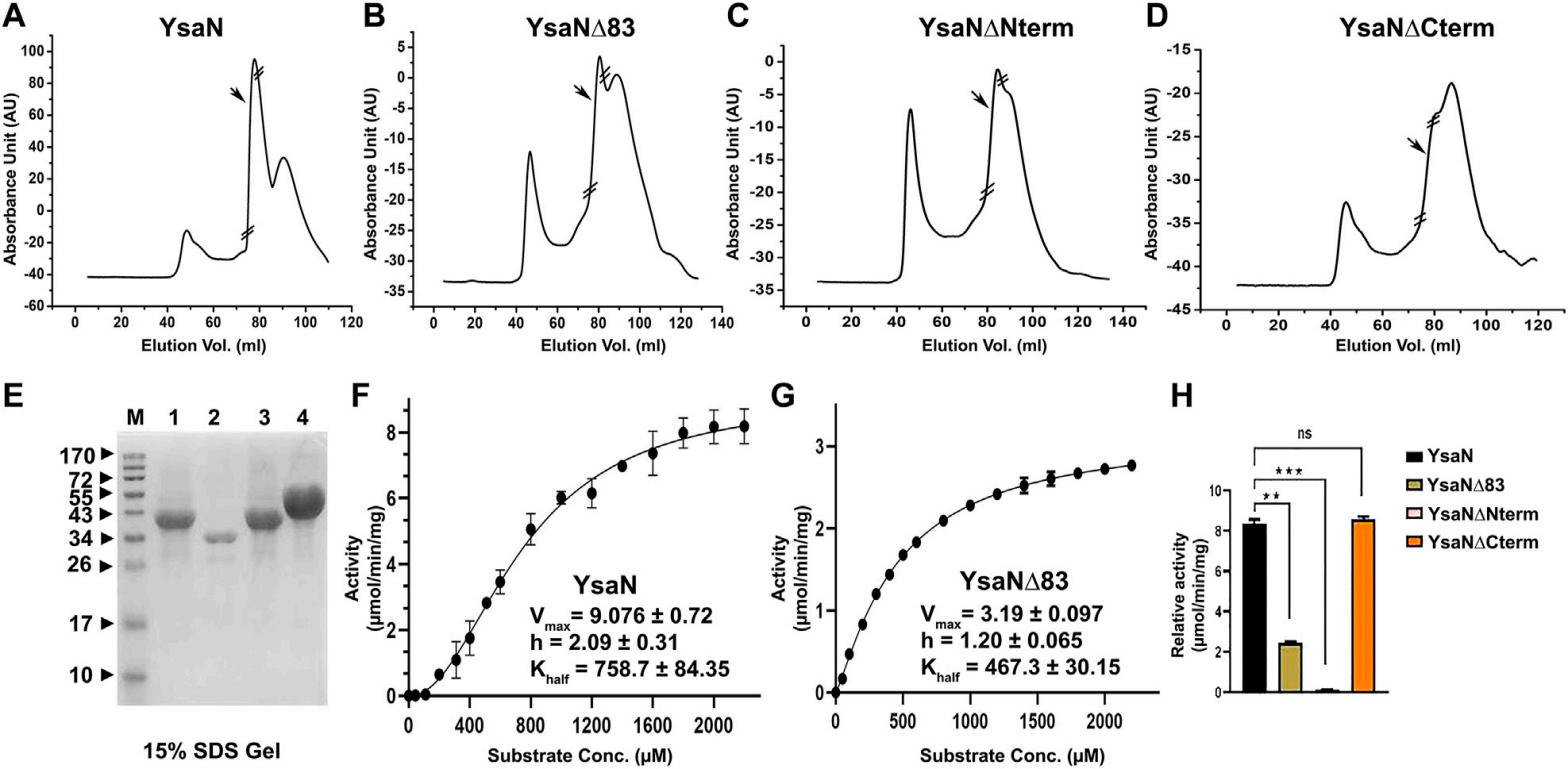
Purification and characterization of YsaN- **(A–D)**—Typical size exclusion chromatography profile of YsaN, YsaN∆83, YsaN∆Nterm, and YsaN∆Cterm respectively using Superdex 16,600 columns. Respective collected peaks are delimited by crossed sections of the chromatograms. **(E)** SDS gel profile of collected peaks from gel filtration (Lane M-Marker, Lane1- Peak from **(D)**, Lane2- Peak from **(C)**, Lane3- Peak from **(B)**, and Lane4- Peak from **(A)**. **(F)**- Standard enzyme assay curve of purified YsaN measuring µmols of phosphate released per minutes per milligrams of protein using the malachite green method for inorganic phosphate estimation, inset showing V_max_, K_half,_ and Hill coefficient values. **(G)**- Standard enzyme curve for YsaN∆83 in terms of µmols of phosphate released per minute per milligrams of protein, inset showing V_max_, K_half,_ and Hill coefficient values. **(H)**- Comparison of relative ATPase activity of purified YsaN to YsaN∆83, YsaN to YsaN∆Nterm, YsaN to YsaN∆Cterm (also please see Supplementary Figure S5A,B for further details of enzyme kinetics of YsaN∆83 and YsaN K166→A and relative assay). Error bars indicate standard deviation in all cases. Statistically significant differences by unpaired Student’s *t*-test are indicated with asterisks (”★★★” means *p* < 0.001; “★★“*p* > 0.01; “ns” means non-significant).

To check the functional activity of all the constructs, malachite- green ATPase assay was performed according to (Lanzetta et al., 1979; Baykov et al., 1988) with slight modification. Briefly, protein in assay buffer (20 mM Tris pH8.0, 100 mM NaCl, 10 mM Mgcl_2_, 1 mM DTT, 0.025% BSA, and 5% glycerol) was prepared sufficiently before performing the experiment. Malachite green- ammonium molybdate reagent mixture was prepared according to (Dey et al., 2018). All the reagents and buffers were filtered using a 0.22 µm syringe filter every time just before use. Varying ATP concentration stock was prepared (0–2,400 µM). Two milliliter of the reaction mixture was prepared by mixing protein at final working concentration of 5 µM for YsaN, and 10 µM for YsaN∆83 in the assay buffer with varying ATP concentrations. 100 µl of reaction sample was taken at four different time points and added to 96 well plate containing 50 µl of the malachite green reaction mixture and incubated for 10–20 min. To stop further change in color development, 100 µl of 34% citric acid (Merck) solution was added to the reaction and incubated for another 30 min at 300 K. Finally, the absorbance was read at 660 nm in Hidex- sense 96 well plate reader. Separate independent experiments were performed in triplicates for YsaN and YsaN∆83 enzyme kinetics. To further compare the activity of different YsaN constructs relative ATPase activity graph was obtained by using a final working concentration of 5 µM for YsaN, YsaN K166→A, and YsaN∆Cterm whereas 10 µM for YsaN∆83 and YsaN∆Nterm respectively (Figure 2H, Supplementary Figure S5). Briefly, in all cases, a standard 2 mM ATP (working) was incubated for up to 40 min in a total of 2 ml reaction volume and 100 µl of reaction sample was taken at eight different time intervals (from 5 to 40 min) and added to 96 well plate containing 50 µl of malachite green- ammonium molybdate reagent mixture as mentioned above. Following a similar assay protocol relative assay was also performed in an assay buffer containing 2 mM EDTA and with the assay buffer containing 5 mM NaF as a control experiment to test the effect of NaF on YsaN activity (Supplementary Figure S5B). Relative ATPase activity experiments were performed in duplicate in an independent experiment. A separate blank for each experiment was generated using assay buffer and ATP (respective concentration) with no protein and the values were subtracted from the experimental results. Inorganic phosphate released was calculated from the phosphate standard curve generated (Supplementary Figure S4) using NaH_2_PO_4_ (Sodium phosphate monobasic, Sigma). The K_half_, V_max,_ and Hill coefficients were observed by plotting the non-linear regression plot in GraphPad prism8 software. The graph was plotted using Origin8 and GraphPad prism 8 software.

### 2.4 Dynamic light scattering experiment

Dynamic light scattering (DLS) was performed using Zetasizer Nano ZS (Malvern Instruments, United Kingdom) with the samples to check mono-dispersity of samples in the presence and absence of various concentrations of ATP in DLS buffer (20 mM Tris pH8.0, 100 mM NaCl, 4mM Mgcl_2_, 5% glycerol and 1 mM DTT). 100 µl of 10–100 µM protein samples with varying ATP concentrations were mixed and incubated for 2–5 min at 298 K. 50 µl of this sample was added in Ultra-Micro Cell ZEN2112 (Malvern Panalytical, United Kingdom), and size intensity distribution was observed. Substrates like ADP, AMP-PNP, and ADP. AlFX (1–3.0 mM range) was also used in the DLS experiment. All the DLS experiments were performed more than three times in all cases with freshly purified samples in independent experiments.

### 2.5 Size exclusion chromatography

Further, the analytical size exclusion chromatography (or gel filtration) method was performed to characterize the oligomeric behavior of YsaN. We used ADP.AlFX (a non-hydrolyzable ATP to ADP transition state analog, refer to Figure 4 **)**, to mimic ATP. YsaN-ADP.AlFX and YsaN∆83-ADP.AlFX complex formation was done according to Rappas et al. (2005) with slight modification. Briefly, 500 µl of approximately 10 µM purified YsaN and YsaN∆83 protein was incubated with 1.5 mM of ADP for 5–10 min at room temperature. To this pre-incubated sample, 1.5 mM AlCl_3_ (working concentration) was added and mixed thoroughly followed by incubation for another 20 min at room temperature. This sample was injected into gel filtration system with pre-equilibrated Superdex 200 H R 10/30 analytical column (GE Healthcare) with 50 mM Tris pH 8.0, 100 mM KCl, 4 mM MgCl_2_, 5 mM NaF, 1 mM DTT and 5% glycerol. NaF in gel filtration buffer was required for maintenance of the AlFX complex (Rappas et al., 2005). The flow rate was kept at 0.5 ml/min at room temperature. All the respective peaks were collected, concentrated, and analyzed by SDS gel electrophoresis (Figure 4F).

### 2.6 Transmission electron microscopy

Negative TEM was performed to visualize YsaN and YsaN∆83 oligomers to a higher resolution. Briefly, approximately 0.6–0.8 µM of purified YsaN and YsaN∆83 was incubated with 1.5 mM ADP for 10 min at room temperature. To this sample 1 mM (working concentration) of AlCl_3_ was added and incubated for another 15 min at room temperature. 3 µl of this sample was loaded onto a freshly glow discharged carbon-coated copper 300 mesh grid and left for 30 s. Excess of the sample was blotted gently using blotting paper. The precaution was taken not to dry the grid completely. Then 5 µl of 1% uranyl acetate solution (Cornelis et al., 1998) was loaded onto the grid and left for 10 s. Excess stain was blotted gently using blotting paper. The staining process was repeated three times. The stained grids were left to air dry at room temperature and stored for viewing purposes. The negatively stained samples were visualized at room temperature using a TALOS L 120C electron microscope (Thermo Fisher). The instrument was operated at 120 kV and the images were captured using a bottom-mounted Flucam and Ceta 16M Camera (35–910 kX magnification range).

## 3 Results

### 3.1 *In- silico* study of YsaN

T3SS ATPase family proteins contain three predicted domains: an N-terminal domain ≈with 100 residues involved in oligomerization and stabilization of the ATPase ring complex, a central ATPase domain, and a C-terminal domain for effector-chaperone complex interaction. In our previous study, we have shown that YsaN is the putative ATPase of Ysa T3SS in *Y. enterocolitica* 8081 (26). To investigate it further we did various *in-silico* studies mentioned here to analyze the YsaN sequence and the nature of its N-terminal domain. Multiple sequence alignment (MSA) of YsaN was conducted using Clustal Omega (Sievers et al., 2011) with the homolog proteins mentioned in Supplementary Table S2. The MSA (refer to Figure 1C; Supplementary Tables S3, S4) shows that the N-terminal domain is less conserved unlike the central domain and the C-terminal domain among different homologs. Further, domain analysis of YsaN using P*fam* (Bateman et al., 2000) suggests the probable presence of ATP synthase alpha and beta subunits signatures at 139–349 amino acids with a bit score of 246.8 and E-value score equals 1.9e-76 (Figure 1B). Secondary structure prediction using PSIPRED 4.0 with YsaN sequence (Jones, 1999) (Supplementary Figure S1B) suggests that the N terminal region primarily consists of *β* strands. Also, Kyte & Doolittle’s hydrophobicity result in ProtScale (Gasteiger et al., 2005) suggests that the N- terminal region (up to 80 amino acids) is primarily hydrophobic (Supplementary Figure S1A **)**. Further a YsaN model was generated using Phyre2 server (Kelley et al., 2015). Phyre2 could generate a YsaN model for 78–430 amino acids only. To generate the full length YsaN homology structure we used MODELLER 9v.11.43 software and for template we used chain D of FliI-FliH crystal structure (PDB ID 5B0O). FliI is fT3SS ATPase in *E. coli* and has 38.28% sequence identity with YsaN (details of model generation process is provided in Section 3.5). Also, using PyMOL both the structures were aligned. The aligned structure has a RMSD value of 1.2Å (Supplementary Figure S10).

### 3.2 Purification and characterization of untagged YsaN

In pursuit of obtaining monomeric soluble YsaN, we tried different YsaN deletions (a random 20 amino acid N-terminal deletion constructs were cloned along with the deletions based on P*fam* domain prediction; Figure 1B), out of which only the constructs mentioned in Table 1 and represented in Figure 1A were used in this study. Since all the YsaN His tagged deletion constructs were insoluble during recombinant expression (data not shown) hence, we cloned YsaN and various deletions in the CPD vector (Table 1) for their soluble expression. One benefit of this CPD fusion was getting untagged protein in soluble form. A typical size-exclusion profile of all the constructs used in this study is shown in (Figures 2A–D **)** followed by their molecular weight analysis by SDS gel (Figure 2E **)**. Also, to check whether CPD fusion resulted in any change or had any effect on oligomeric behaviour of YsaN in solution we compared the size exclusion profiles of C- terminal His tagged YsaN (cloned in *pET*22b vector) with untagged YsaN obtained after removal of C- terminal CPD-His tag. Both YsaN- His and YsaN (untagged) elute at the same elution volume (Supplementary Figure S3). To estimate the functional efficiency of different constructs concerning YsaN enzyme kinetics their ATPase activity was studied by the Malachite green ATPase assay method. YsaN (untagged) shows kinetic parameters–V_max_ value of 9.07 ± 0.72 µmol/min/mg, K_half_ value of 758.7 ± 84.35 µM, and Hill coefficient value of *h* = 2.09 ± 0.31 (Figure 2F), comparable to our previous study on YsaN (N-terminal His-tag) (Chatterjee et al., 2013). YsaN∆83 shows reduced ATPase activity whereas, in the case of YsaN∆Nterm, the activity was barely detectable and YsaN∆Cterm (a C terminal deletion variant of YsaN), shows ATPase activity comparable to YsaN (refer to Figure 2H, Supplementary Figure S5A). These observations suggest that the ATPase activity of YsaN is controlled by its N terminal region. Further to investigate the cooperative nature of YsaN∆83 we perform enzyme kinetics for YsaN∆83 (at 10 µM working concentration, also refer to Figure 2G). YsaN∆83 has a reduced V_max_ of 3.19 ± 0.09, K_half_ value of 467.3 ± 30.15, and a hill coefficient value of *h* = 1.20 ± 0.06. The hill coefficient value obtained for YsaN∆83 suggest that YsaN∆83 bears cooperative nature.

### 3.3 Oligomerization behaviour of YsaN and YsaN∆83

**FIGURE 3:**
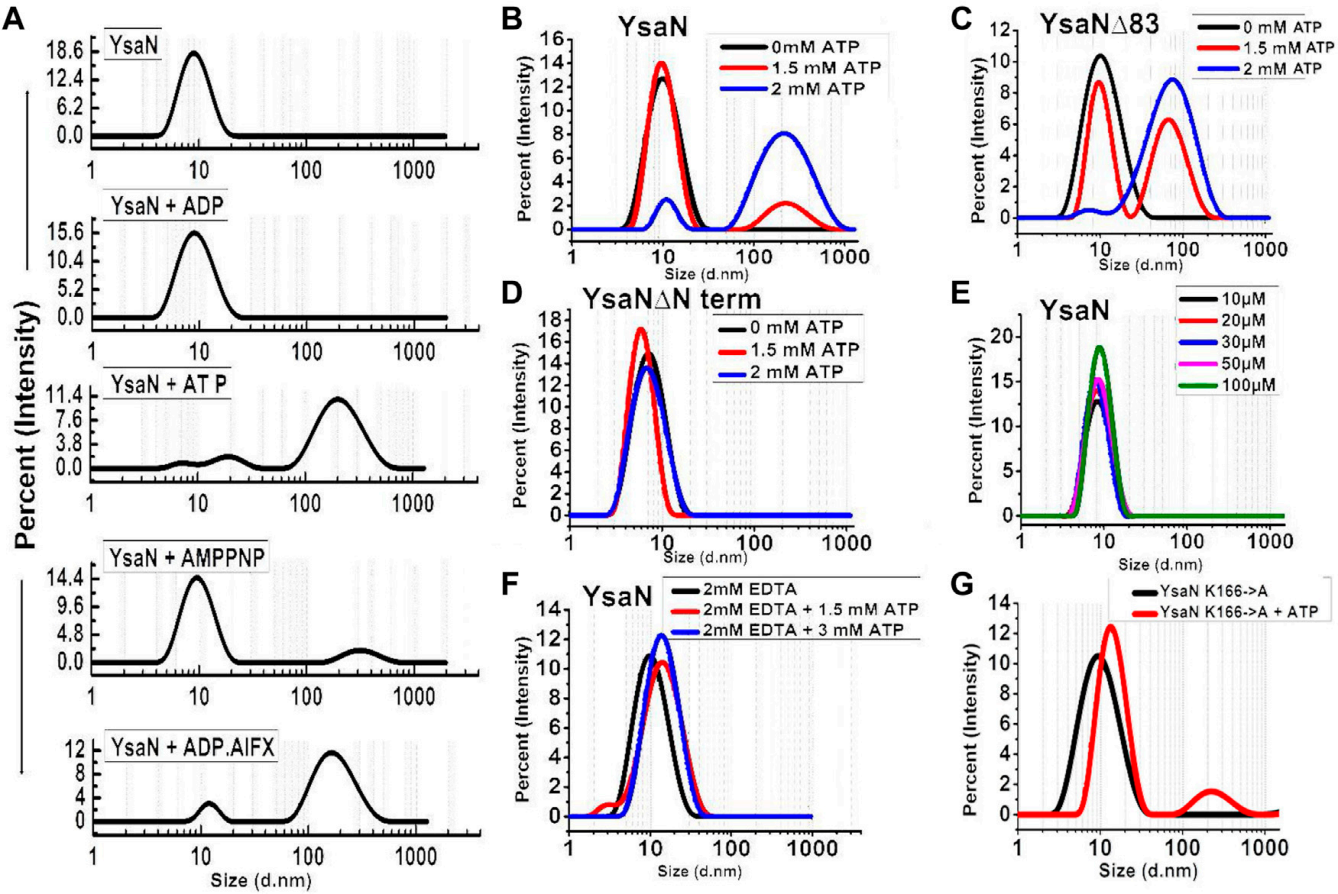
Dynamic light scattering study of YsaN oligomerization—**(A)**- Representation of size intensity profile of YsaN and in the presence of substrates like 2 mM ADP (product), 2 mM ATP (substrate), 2 mM AMPPNP (a non-hydrolyzable ATP analog), and 1.5 mM ADP.AlFX (an ATP to ADP + Pi transition state analogue). **(B**–**D)-** Substrate (ATP) concentration-dependent oligomerization of YsaN, YsaN∆83, and YsaN∆Nterm respectively demonstrating the role of YsaN N- terminal domains in YsaN oligomerization. **(E)-** Protein concentration-dependent DLS study of YsaN. **(F)-** Size intensity profile of YsaN in presence of 2 mM EDTA demonstrating the role of Mg^2+^ dependent catalysis of ATP to ADP + Pi and its role in oligomerization. **(G)-** Size intensity profile of YsaN Walker-A lysine mutant YsaN K166→A in the absence and presence of 2 mM ATP.

The cooperative nature of YsaN and YsaN∆83 prompted us to check the substrate-dependent oligomerization nature of YsaN and YsaN∆83. ATP (substrate), ADP (product), AMP-PNP (non-hydrolysable ATP analog), and ADP.AlFX (ATP to ADP transition state analog) were tested for their role in the oligomerization behaviour of YsaN and was studied by DLS, represented in Figure 3. Figure 3A shows the oligomerization behaviour of YsaN where a single monodisperse peak of is visible for YsaN only, YsaN in presence of 2 mM ADP, and YsaN in presence of 2 mM AMP-PNP. However, the peak got shifted toward right indicating formation of higher order oligomer in the case of YsaN incubated with 2 mM ATP and 1.5 mM ADP.AlFX. Further DLS was also performed in an ATP concentration-dependent and protein concentration-dependent manner to evaluate the oligomeric behaviour of YsaN. Figure 3E shows that increasing protein concentration does not have any effect on YsaN oligomerization whereas increasing ATP concentration has a significant role in its oligomerization (Figure 3B). Similar substrate-dependent behaviour was also observed in the case of YaN∆83 (a partially active construct of YsaN) (Figure 3C) while the oligomerization behaviour was absent in the case of YsaN∆Nterm (a non-functional YsaN construct) (Figure 3D). From Figure 3A it was also observed that AMP-PNP (an ATP analog) was unable to promote oligomerization of YsaN while ADP.AlFX [also an ATP analog, which mimics ATP to ADP transition state (Schumacher et al., 2004; Chen et al., 2007)] was capable of inducing oligomerization. To investigate this behaviour of YsaN we ask the question that whether ATP binding is the key to induce YsaN oligomerization or YsaN oligomerization is a result of the active catalysis of ATP. To evaluate this, we created a Walker-A lysin mutant YsaN K166→A which is a non-functional mutant (Supplementary Figure S5B) of YsaN, and observed its oligomerization behaviour by DLS in a similar way (Figure 3G). In another experiment, we treated YsaN with 2 mM EDTA (during its purification) to quench the Mg^2+^ ion, to prevent Mg^2+^ dependent ATP hydrolysis. YsaN EDTA treated sample was unable to oligomerize even in presence of 3 mM ATP (Figure 3F). In both experiments it was observed that YsaN oligomerization requires active catalysis. Collectively, YsaN oligomerization and its activity depend on the N terminal domain and is an ATP concentration-dependent event and requires active catalysis of ATP to ADP and P*i.*

### 3.4 Formation of YsaN higher-order oligomer by YsaN and YsaN∆83

In the previous section, we observed and identified YsaN∆83 a YsaN construct that is functionally active but, with lesser strength. It is also observed that the formation of YsaN oligomers is very much dependent on the active catalysis of ATP. So, active catalysis and oligomer formation are very much controlled by a specific region of the N-terminal part of YsaN. From the literature, we know that AAA + ATPase like YsaN is capable of forming various higher-order oligomers like hexamer, dodecamer, and higher-order aggregates. So far, the hexamer is the most stable variant of such ATPase complex which has been observed in various *in-situ* studies.

**FIGURE 4:**
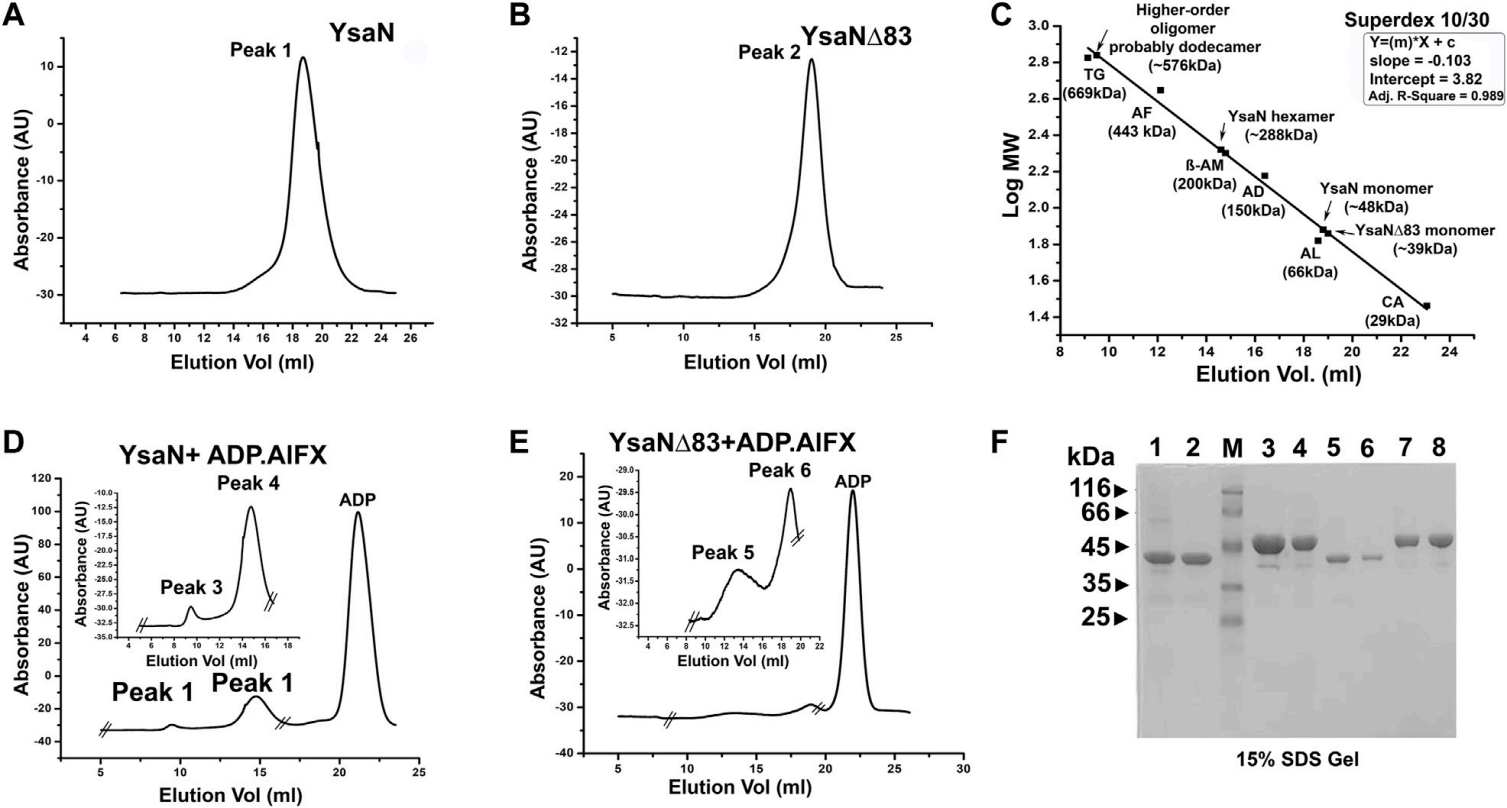
Analytical size exclusion chromatography profile of YsaN and YsaN∆83 using Superdex 10/30 column**- (A)** and **(B)**- size-exclusion profile of purified monomeric YsaN and YsaN∆83 respectively. **(C)**- molecular weight standard curve for Superdex 10/30 column representing elution profiles of possible oligomeric forms of YsaN and YsaN∆83. (TG = Thyroglobulin- 669 kDa, AF = Apoferritin 443 kDa, β-AM = β- Amylase- 200 kDa, AD = Alcohol dehydrogenase- 150 kDa, AL = Albumin- 66 kDa, CA = Carbonic anhydrase- 29 kDa). **(D)** and **(E)**- size-exclusion profile of YsaN and YsaN∆83 in presence of 1.5 mM ADP.AlFX in the buffer containing 5 mM NaF, inset showing peaks corresponding to probable dodecamer and hexamer respectively in the zoomed image delimited by crossed section in the full chromatogram. **(F)**- SDS gel profile of the purified sample and peaks collected during size exclusion chromatography steps. Lane 1 and Lane 3 represent purified YsaN∆83 and YsaN form Superdex 200 16/600 size exclusion chromatography column respectively. Lane 2 (YsaN∆83) and Lane 4 (YsaN) correspond to peak 2 of **(B)** and peak 1 of **(A)** respectively. Lane 3 represents the marker, and Lane 5 and 6 represent the peak 6 and peak 5 in **(E)** respectively. Lane 7 and 8 represent peak 3 and peak 4 in **(D)** respectively.

The role of the N-terminal region in YsaN oligomerization and its ATPase activity was further studied through estimation of the apparent molecular weight by analytical size exclusion chromatography (Superdex 200 H R 10/30 column) of the oligomeric complexes of YsaN and YsaN∆83 in the absence (Figures 4A,B respectively) and in presence of ADP.AlFX (Figures 4D,E respectively). Apparent molecular weight estimation was done by comparing the elution profiles with the molecular weight standard curve represented in Figure 4C. YsaN-ADP.AlFX, complex eluted at ≈14 ml and ≈10 ml which corresponds to the molecular weight of ≈288 kDa (hexamer), and ≈576 kDa (probably dodecamer) respectively**.** A similar experiment was performed with YsaN∆83-ADP.AlFX a peak corresponding to hexamer (molecular weight of ≈234 kDa) along with an un-oligomerized peak (Figures 4B,E respectively) was observed. The respective peaks were collected and analyzed by SDS gel electrophoresis (Figure 4F).

**FIGURE 5:**
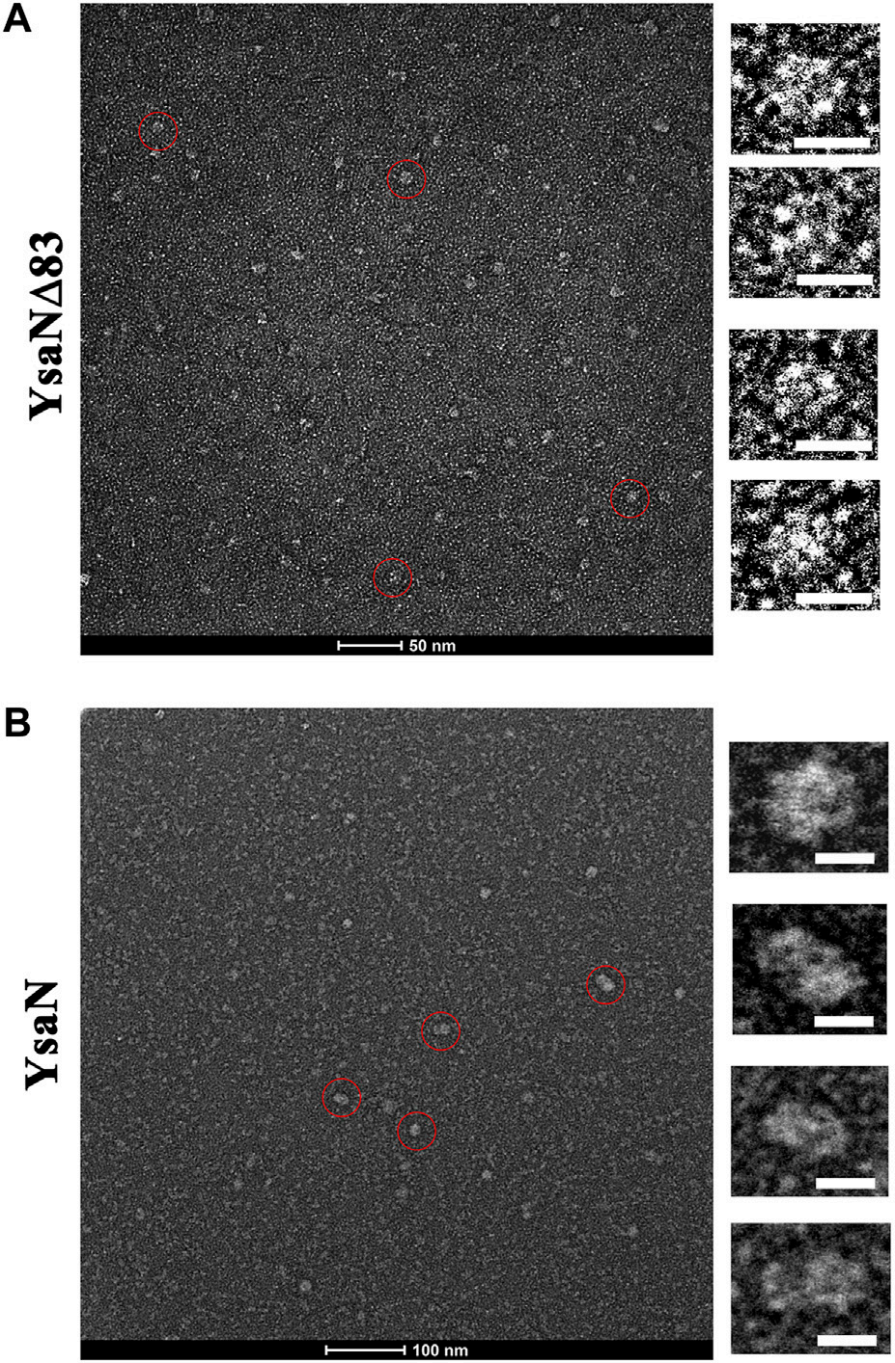
Negative TEM image of YsaN and YsaN∆83 oligomer in presence of ADP. AlFX. **(A)** YsaN∆83 oligomer. The Zoomed image represents an approximately 10 nm hexamer ring assembly of particles marked by the red circle in a different orientation. **(B)** The YsaN higher-order oligomer complex. The zoomed image represents particles marked by the red circle in a different orientation having a size of approximately 15–20 nm in different orientations representing probable dodecamer. The prepared sample grids were stained with 1% uranyl acetate. The white color scale bar represents 10 nm in all cases.

Following the previous sections, YsaN and YsaN∆83 have catalytic activity and they can form functional oligomeric structures in solution, we extended our work to visualize these oligomers in greater detail. We used negative-TEM imaging techniques to visualize these oligomers. Similar experimental conditions were chosen for these imaging studies which have been used in DLS and gel filtration studies. YsaN and YsaN∆83 was viewed in absence and in presence of ATP and ADP.AlFX. While viewing YsaN and YsaN∆83 oligomers in negative-TEM using ATP and ADP.AlFX, we were able to get better images of oligomer in the presence of ADP.AlFX as compared to ATP. In the case of YsaN∆83, we were able to visualize the hexameric ring structure of YsaN∆83. It should be noted here that ADP.AlFX has been reported to stabilize the oligomeric structure of similar ATPase in earlier studies and has been used extensively to obtain high-resolution TEM and Cryo-EM structures (Majewski et al., 2019). In the case of YsaN∆83-ADP.AlFX, the observed particle size of ≈10 nm representing a hexamer formation with clear sixfold symmetry was observed (Figure 5A). The particle size (10 nm) of the hexamer as observed in TEM images are similar to the previously reported size of 10 nm among the three-dimensional structures of hexameric ATPases. Whereas, in the case of YsaN-ADP.AlFX the particle size of the oligomer observed was greater than 10 nm and was observed as ≈ a 15–20 nm structure in different orientations (Figure 5B). The presence of particle size greater than 10 nm indicates the presence of a higher oligomeric form in the YsaN complex as compared to YsaN∆83.

The presence of particles having more than 10 nm in size indicates the presence of a higher oligomeric form in the YsaN complex, as compared to YsaN∆83. We assume this higher-order oligomeric form to be probable YsaN dodecamer. It is also notable that the presence of a few other intermediate oligomers in the YsaN∆83-ADP.AlFX in TEM image suggests that the formation of the hexamer complex was weak which may be a result of the deletion of the N-terminal 83 residues which may be required for stable hexamer ring formation post oligomerization. Collectively YsaN∆83 is capable of hexamer formation independent of 83 N-terminal residues.

### 3.5 Homology model structure of YsaN oligomer

**FIGURE 6:**
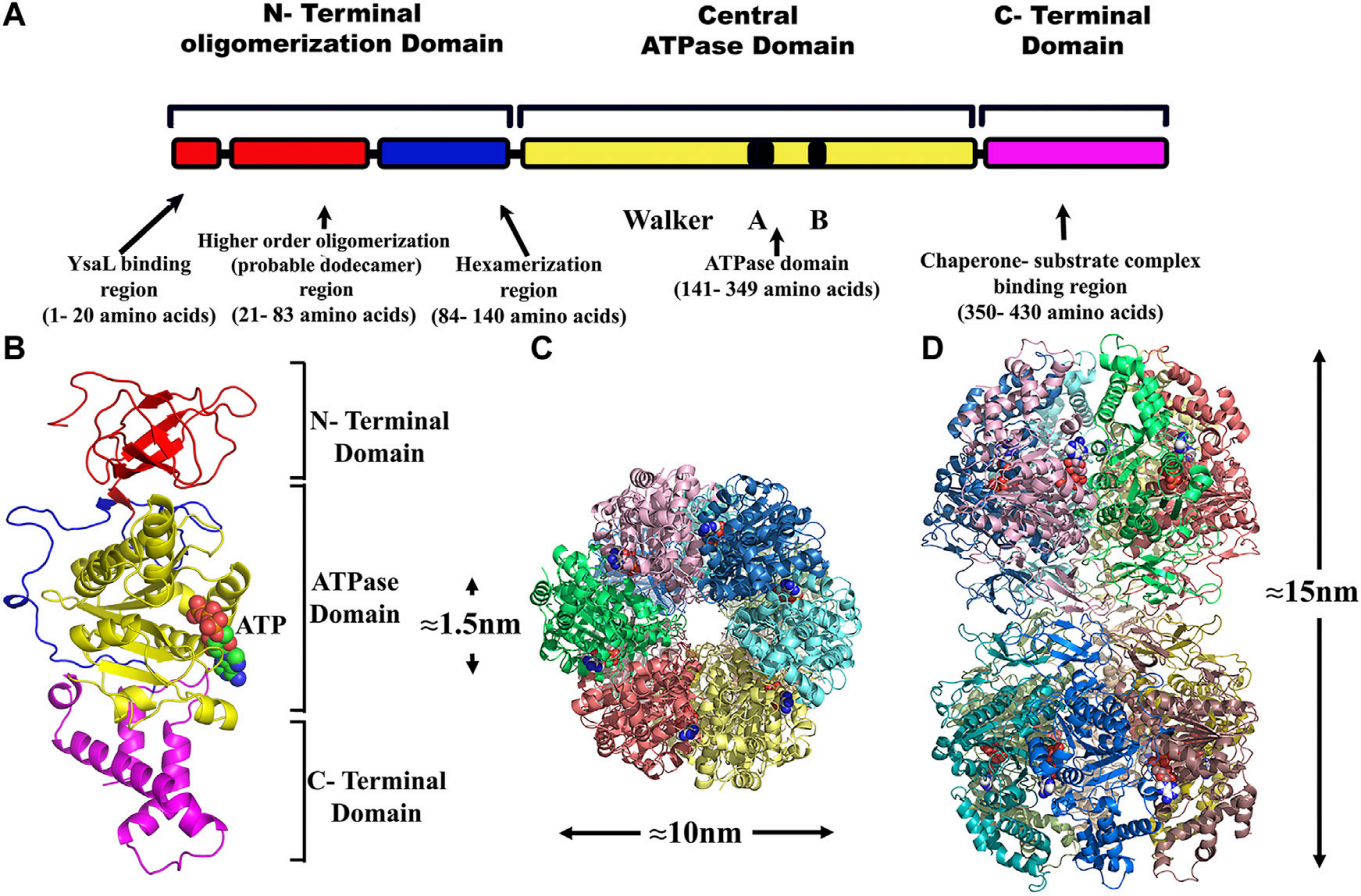
Structure modeling of YsaN and YsaN dodecamer complex- **(A)**- Linear representation of different predicted domains in YsaN. **(B)**- The model structure of YsaN is constructed on basis of FliI- FliH crystal structure (flagella T3SS ATPase structure PDB ID 5B0O). **(C,D)**- Model of probable dodecamer build by stacking two homo-hexamers through their N terminal faces following docking study. C- top view, **(D)**- side view.

Since the only full-length crystal structure of FliI (fT3SS ATPase) is present to date, hence, to generate the monomeric homology model, Chain D was chosen (having sequence identity of 38.28% with YsaN) from the crystallographic structure of FliI–FliH complex (PDB Id: 5B0O). The 3D-structure prediction of YsaN was done using the homology modeling software package MODELLER 9v.11.43 (Webb and Sali, 2014). Alignment between template and target was performed by Clustal Omega (https://www.ebi.ac.uk) (Sievers et al., 2011). A few manual improvements were done in alignment files and an in-build python script. These manual improvements of PDB files was done using Coot software (Emsley et al., 2010) to remove the clashes. Finally, at least five models were taken from each modeling procedure. The best model was then selected based on the Discrete Optimized Protein Energy (DOPE) score (Supplementary Figure S9; Supplementary Table S5). The different predicted domains of YsaN were represented by a linear map in **(** Figure 6A). The final model generated was viewed using PyMOL software (DeLano, 1874) **(** Figure 6B **)** and subsequently, the hexameric form was built by fitting the monomeric structure into the hexamer of the F1 ATPase (PDB Id: 4XD7) by the use of PyMOL software. Again, some manual improvement of a hexameric text file was done. After generating the model some unnecessary text appears in the PDB text file, which breaks chain continuity and cause a problem during visualization in softwares like vmd, PyMOL etc. To solve this problem, we have to manually delete such kinds of unnecessary text from PDB text file. Using this hexameric text file the dodecameric form of the YsaN (N terminal 20 amino acids deleted) structure was generated by the ClusPro server (Kozakov et al., 2013; Kozakov et al., 2017). Also, Coot was used manually to remove the clashes caused by close contacts of amino acid side chain. These clashes were removed by rotating side chains to a non-clashing rotamer. Structural evaluation and stereochemical analysis of modeled protein were checked by various bioinformatics tools and software packages such as RAMPAGE, ERRAT, and Verify3D. Molecular docking was performed by an automated docking tool, Auto Dock (Trott and Olson, 2010). The software was used for the binding of small molecules (ATP) with a structural model of its known three-dimensional receptor protein (YsaN). It uses the genetic algorithm for conformational search and it is regarded as a popular method to study docking. The technique combines simulated annealing for conformation searching with a rapid grid-based method of energy evaluation. During the docking simulations, the inhibitors were regarded as flexible and subjected to energy minimization and scoring system based on force-field energy scoring function. By the use of PyMOL, the hexameric structure of YsaN was generated by fitting into F1 ATPase (PDB Id: 4XD7). Some manual improvements were done in the hexameric form of YsaN to generate a dodecameric structure. The dodecameric structure was built using the ClusPro server (Kozakov et al., 2017) *via* three computational steps: 1) rigid-body docking, 2) RMSD based clustering of the lowest energy structures, and 3) the removal of steric clashes by energy minimization. Docking with each energy parameter set results in ten models defined by centers of highly populated clusters of low energy docked structures which were picked up for final model generation. Based on the above study, the YsaN hexamer- hexamer docked model was generated to represent the pre assumed dodecameric double-ring structure. The double ring structure was modeled by docking the N- Terminal residue faces of two hexamers **(** Figures 6C,D). The dodecamer model of YsaN has approximately 15 nm length along with the stacked interface and approximately 10 nm diameter along each hexameric ring. A clear approximately 1.5 nm pore is visible from the top of the dodecamer structure. Following a similar protocol, YsaN∆83 homology model was built from FliI template using MODELLER software. Based on their DOPE score, five models were chosen. The best model was utilized to construct a YsaN83 hexameric structure. YsaN83 hexamer was constructed using PyMOL software by fitting YsaN83 into the F1 -ATPase (PDB ID 4XD7) hexamer structure (Supplementary Figure S11).

## 4 Discussion

T3SSs are complex nano-syringe-like structures used by many pathogenic bacteria to inject effector toxins directly into the host cell cytoplasm (Coburn et al., 2007; Buttner, 2012). Efficient unfolding and translocation of an effector require active interaction of the effector-chaperone complex with the highly conserved T3SS ATPases inside (Akeda and Galan, 2005) bacterial cytoplasm. The oligomeric form of these ATPases is the most active form present at the export gate. In previous studies, hexamer and dodecamer have been reported as functional oligomeric forms of these ATPases (Pozidis et al., 2003; Andrade et al., 2007; Burgess et al., 2016). In *Y. enterocolitica*, YscN (a putative energizer of *ysc-yop* T3SS for Yop secretion) has been suggested to be activated through oligomerization and work as a hexamer (Woestyn et al., 1994). However, the true nature of the activation and formation of the different oligomers is still poorly understood. Here, we report the existence of a higher-order oligomer (probable dodecamer) as well as a hexamer, as the functional oligomeric form of ATPase YsaN from *Y. enterocolitica ysa-ysp* T3SS. In our previous *in-vitro* study, the possible dodecamer has been identified as the most active form of YsaN over the weakly active monomeric form (Chatterjee et al., 2013). Based on different biochemical and biophysical methods, the present study analyzes the mechanism of formation of different functional oligomers of YsaN and its correlation with the N-terminal region.

P*fam* domain analysis of YsaN protein sequence and its sequence homology with the identified and well characterized T3SS ATPase from *Shigella flexineri* (Spa47), *E. coli* (EscN), *Pseudomonas syringae* (HrcN), flagellar ATPase FliI and with the β-subunit of F_1_-F_0_ ATPase suggest that YsaN is also an T3SS associated ATPase of *Y. enterocolitica*. Recombinant expression of YsaN N-terminal His resulted in mixed YsaN population predominantly higher oligomeric form. Also, any deletion of YsaN render it insoluble and difficult to purify (Chatterjee et al., 2013). We have developed an expression system which allowed expression of untagged YsaN and soluble expression of any YsaN deletion construct in its native monomeric form. The ability to purify all the YsaN deletion construct in soluble monomeric form resulting from CPD fusion using CPD-vector has been crucial for this study (Shen et al., 2009). This allows us to capture the real-time oligomerization behaviour of YsaN (i.e., transition from monomer to hexamer and hexamer to higher-order oligomer as stable oligomeric form) and the involvement of its N-terminal region. Enzyme kinetics study of YsaN (untagged) obtained here are comparable to previous studies and other homologs (Andrade et al., 2007; Chatterjee et al., 2013; Case and Dickenson, 2018). Also, similar ATPase activity observed for YsaN and YsaN∆Cterm suggests that YsaN ATPase activity is independent of its C-terminal domain. Whereas, the absence of ATPase activity in YsaN∆Nterm and the presence of activity in YsaN∆83 (however reduced as compared to YsaN) suggest that YsaN activity depends on its N-terminal domain (1–140 residues). Also, the presence of activity in YsaN∆83 (an N-terminal deletion variant of YsaN) breaches the canonical thinking of loss of activity of N-terminal deletions (up to ≈79 residues) variants of homolog ATPases (Burgess et al., 2016). The presence of the cooperative nature of YsaN∆83 (*h* = 1.20 ± 0.06) also reveals the two-step cooperative behaviour of YsaN activation. This indicates that the ATPase activity of YsaN requires both N-terminal regions: 1–83 and 84–140 residues in an independent manner. Hence, we propose that the complete activation of YsaN requires both of its N terminal regions including 1–83 and 84–140 amino acid residues.

DLS study suggests that YsaN changes its form upon ATP binding as a result of its oligomerization. YsaN in presence of AMPPNP and ADP.AlFX (both of which are ATP analog), shows different oligomerization behaviour in the DLS study. This difference in oligomerization behaviour may be a result of the requirement of active catalysis of ATP for YsaN oligomerization and not just ATP binding. This hypothesis was supported by YsaN 166K→A site-directed mutation study. In another experiment, removing Mg^2+^ ions to stop magnesium-dependent hydrolysis of ATP by using EDTA also resulted in the loss of oligomerization and activity of YsaN. Hence, we propose that the binding of ATP to YsaN is not the prime factor responsible for its oligomerization. Instead, the active catalysis of ATP to ADP + P_i_ may be the prime factor for YsaN oligomerization. This is similar to the observation in the case of F_1_F_o_- ATPase suggesting the idea of a universal mechanism in these molecular AAA + ATPases across their diverse roles inside the cell (Martin et al., 2014).

From Figures 3B,C, it was observed that both YsaN and YsaN∆83 have the oligomerization tendency dependent on increasing ATP concentration. Also, the estimated size of the oligomer in the case of YsaN is larger than the oligomer formed in the case of YsaN∆83. Oligomerization of T3SS ATPase of *Shigella*, Spa47 has been shown to be dependent on protein concentration (Andrade et al., 2007), whereas YsaN oligomerization occurs in an ATP (substrate) concentration-dependent manner. To investigate this, we have performed DLS of YsaN in a concentration-dependent manner. It was observed that by increasing the concentration of YsaN, the formation of higher-order oligomers was absent (Figure 3E). Conclusively, YsaN oligomerization is a substrate (ATP) concentration-dependent event and requires active catalysis of ATP to ADP + P*i*. It is also proposed that YsaN∆83 bears the oligomerization behavior upon ATP binding and hydrolysis unlike, other homolog ATPases N-terminal deletion variants, which was previously considered non-functional. Further, to estimate the apparent molecular weight of the oligomeric complexes we performed analytical size exclusion chromatography (Figure 4). YsaN treated ADP.AlFX shows the presence of two complex forms corresponding to YsaN oligomer equivalent to probable dodecamer and hexamer corresponding to a molecular weight of approximately 576 and 288 kDa respectively. Whereas, ADP.AlFX treated YsaN∆83 shows only a single complex of approximately 234 kDa corresponding to hexameric form along with an un-oligomerized peak. Also, we observed the oligomers in negative-TEM. In TEM study the observed size of YsaN and YsaN∆83 oligomer was seen as ≈15–20 nm (Figure 6B) and ≈10 nm (Figure 6A) respectively. The observed sixfold symmetry of the YsaN∆83 hexamer in TEM suggests that YsaN is capable of forming hexamer independent of N terminal 1–83 amino acid residues when bound to ADP.AlFX. This property of hexamer formation in T3SS ATPase with N- terminal 83 residues deleted has been observed for the first time. While in the case of YsaN, the higher observed size of the particles suggests the presence of a higher-order oligomeric form greater than hexamer which we propose as the YsaN probable dodecamer. In many attempts, during TEM sample preparation of YsaN oligomer, few of the particles greater than 20 nm were also observed which is certainly non-specific aggregates resulting from binding of ADP.AlFX **(** Supplementary Figure S6) which was absent in the case of YsaN∆83 **(** Supplementary Figure S7 **)**. It seems difficult to control the *in-vitro* oligomerization, avoiding the formation of non-specific aggregates for YsaN. It is to mention that decreasing protein concentration limits the formation of aggregates resulting in fewer particles observed in TEM images. The 10 nm size of the YsaN∆83 hexamer reported here is similar to the observed size of 10 nm for the homo hexameric T3SS ATPase in *E. coli* (Majewski et al., 2019). Conclusively, the N- terminal 1–83 residue of YsaN is crucial for higher-order oligomer formation while the formation of hexamer is independent of this region and requires 84–140 residues. The N-terminal 1–20 residues of YsaN are required for YsaL interaction (Chatterjee et al., 2013). The formation of higher-order oligomer was absent in the case of YsaN∆83. Also, it has been observed that the YsaN∆Nterm is incapable to form oligomer. The above observed results guided us to build a dodecamer model by stacking two hexamers through their N terminal faces (Figures 6C,D). A clear pore of about ≈1–1.5 nm can be easily seen confirming the possibility of N-to-N terminal stacking between two hexamers. Also, bioinformatic sequence analysis of YsaN shows the hydrophobic nature of the predicted beta-strand of the N-terminal domain primarily from 21 to 83 amino acid residues (Supplementary Figure S1). The hydrophobic nature of the predicted beta-strand of the N terminal domain of YsaN might play a crucial role in higher-order oligomer formation as shown in our model of YsaN hexameric complex surface topology map (Supplementary Figure S8 **)**. Hence, there exists the possibility that the formation of the higher-order oligomer (possible dodecamer) may take place by stacking two homo-hexamers by their N- terminal faces. Also, the dodecamer complex might be stabilized primarily by the hydrophobic interaction between N-terminal faces of two independent hexamers. We also propose that the formation of the most active form of YsaN oligomer proceeds in two distinct steps i.e., beginning with the formation of hexamer followed by the formation of higher-order oligomer depending on the ATP concentration.

Significant advancement in the knowledge has been achieved to date about the structure and mechanism of functionally least active monomeric and most active oligomeric form (hexamer or dodecamer) of T3SS ATPases. Yet, no study has been able to answer exactly which active oligomeric structure is required for substrate unfolding and translocation through the T3SS needle complex. The presence of hexamer ATPase ring complex at the T3SS export gate is widely accepted, and also has been observed in *in-situ* cryo-EM structures available to date. Whereas existence of hexamer and dodecamer ATPase complex during *in-vivo* and *in-vitro* studies (where the dodecameric form shows maximum activity) creates ambiguity and confusion about their role. Also, there is lacking of study addressing the exact mechanism of oligomerization among these highly conserved proteins. In the present study, we demonstrate the mechanism of formation of oligomers giving insight into the oligomeric nature of such T3SS ATPase, YsaN as an example. However, the exact mechanism of how these ATPases perform the unfolding and translocation of effectors is still not known. According to Akeda and Galan (2005), an appealing model mechanism among these T3SS ATPases similar to other AAA + ATPases is that the effector chaperone complex docks to such ATPase ring complex while unfolding the effector by threading them through the pore of the ATPase ring complex. The effector docking C- terminal side of the ATPase ring complex faces away from the cytosol towards the needle complex (Imada et al., 2016; Majewski et al., 2019). The hexamer model of ATPase at the export gate may result in possible steric hindrance for proper and free docking of the chaperone effector complex to the C-terminal end of the ATPase ring complex. Instead, the proposed probable dodecameric ATPase ring complex, if present at the active T3SS removes the possibility of steric hindrance and allows free access for docking of chaperone effector complex to the ATPase ring complex towards the cytosolic C- terminal side. The other C-terminal side would be freely accessible for interacting with the positive regulator (SctO) linking it to the membrane component SctV towards the membrane side as has been reported in earlier studies (Zarivach et al., 2007; Kato et al., 2015; Majewski et al., 2019). Another aspect of the proposed dodecamer ATPase model also allows the possibility that unfolded effectors can pass through the narrow pore of the ATPase ring complex during the secretion process similar to the other AAA + protein translocases (Akeda and Galan, 2005). It is worth mentioning that the presence of higher order oligomer (probable dodecamer) at the export gate of T3SS has not been found in any *in-situ* experiment till date. While the presence of hexamer ATPase ring complex has been observed in recent *in-situ* Cryo-EM structure of *Salmonella* T3SS by Hu et al. (2017). The absence of the probable dodecamer ATPase ring complex observed in *in-situ* Cryo-EM structures suggests the possibility that it may be required only during engagement with the effector chaperone complex representing the secretion competent phase of T3SS. It may be noted that whether unfolded effector passes through the ATPase pore or not is still a matter of debate.

These highly conserved T3SS ATPases plays a crucial role in effector secretion and may be a significant target for drug development. To reveal the exact functioning of T3SS it is necessary to understand the role of such ATPases in T3SS regulation. The work reported in this study involving characterization of nature of YsaN oligomerization will provides a foundation for expanding our knowledge to understand T3SS regulation by these ATPases. Overall, the present study suggest that YsaN is an oligomerization activated T3SS ATPase in *Y. enterocolitica.* Also, formation of YsaN oligomer depend on active catalysis of ATP and the whole process of YsaN oligomerization and activation can be summed up as a two-step cooperative kinetic process involving formation of YsaN hexamer followed by formation of higher order oligomer. However, we could not establish the exact oligomeric nature of the most active form of YsaN higher order oligomer in the present study. Eventually, further experiments are necessary to explore the true mechanism of oligomeric ATPase ring complexes in T3SS regulation in *Yersinia enterocolitica* and other pathogenic bacteria.

## Data Availability

The original contributions presented in the study are included in the article/Supplementary Material, further inquiries can be directed to the corresponding author.

## Abbreviations

AAA + ATPases, ATPase associated with various cellular activities; ADP- AlFX, ADP aluminum fluoride complex; AMPPNP, Adenylyl- imidodiphosphate, CPD, cysteine protease domain; DTT, Dithiothreitol; EDTA, Ethylenediaminetetraacetic acid; IPTG, Isopropyl β- d-1, thiogalactopyranoside; NaF, Sodium fluoride; PDB, Protein data bank; PMF, Proton Motive Force; RMSD, Root mean square deviation; RPM, rotation per minute; SDS, Sodium dodecyl sulphate.
