# Supplementary material for: Delineating specific regions of N- terminal domain of T3SS ATPase YsaN of Yersinia enterocolitica governing its different oligomerization states
Source: Front Mol Biosci. 2022 Sep 8;9:967974. doi: 10.3389/fmolb.2022.967974 (PMC9493007; doi:10.3389/fmolb.2022.967974)
Supplement: Supplementary file 1 [file DataSheet1.pdf]

Supplementary material

Table of Contents: **Page No.**

Table S 1: List of primers used in this study………………………………………………...……………….. S2

Table S 2: List of Proteins used for Multiple Sequence alignment………………………................................ S2

Figure S1: Bioinformatic analysis of YsaN ……………………………………………………………………. S3

Table S 3 & S 4: Percent identity table of N terminal and C-terminal based on sequence alignment………..... S4

Figure S2: Molecular weight standard curve for Superdex 16/ 60 column…………………………………...… S5

Figure S3: Effect of CPD fusion on the oligomeric state of YsaN………………........……………………...... S5

Figure S4: Phosphate standard curve for Malachite green assay…………………….…………………….…..... S6

Figure S5: Relative activity assay…………...……………………..………………………………….…………. S6

Figure S6: Negative TEM image of YsaN oligomer complex with ADP.AlFX……………………..……..... S7

Figure S7: Negative TEM image of YsaNΔ83 oligomer complex with ADP.AlFX….……………..…....….........S8

Figure S8: Surface map of YsaN hexameric complex…………………………………………...…..……. S9

Figure S9: Representation of all five aligned structures generated by MODELLER software………………… S10

Table S 5: Table representing the variance in the DOPE score of MODELLER generated YsaN models and the RMSD of aligned models with model 5…………………………………………………………… S10

Figure S10: Representation of aligned structures generated by MODELLER software and Phyre2 server.…..… S11

Figure S11: Homology model of YsaNΔ83 monomer and hexamer…………………………………….….… S12

Table S 1: List of primers used in this study			
**Gene/ construct name**	**Forward (5'- 3')**	**Reverse (5'- 3')**	**Reference(s)/ Source**
YsaNCPD	GGGAATTC**CATATG**AATCTCT TTG ATAGCTGT	CG**GGATCC**GAGTTTGCC AGCTC	IDT Technologies/ This study
YsaNΔ83CPD	GGGAATTC**CATATG**ATGGGAG AA CATCTG	CG**GGATCC**GAGTTTGCC AGCTC	GCC Biotech/ IDT Technologies / This study
YsaNΔNTerm CPD	GGAATTC**CATATG**GGGGTCAG GG CAATTG	CG**GGATCC**GAGTTTGCC AGCTC	GCC Biotech/ This study
YsaNΔCTerm CPD	GGGAATTC**CATATG**AATCTCT TTG ATAGCTGT	CG**GGATCC**GAAGTCACTTTC GAAAAC	GCC Biotech/ This study
YsaN K166→A	GCGGCCGGTAGCGGCGCAACC TCCCTGATGAGC	GCTCATCAGGGAGGTTGCGC CGCTACCGGCCGC	GCC Biotech/ This study
*Note- Restriction site highlighted in bold letters.			

Table S 1: List of primers used in this study.

Table S 2: List of Proteins used for Multiple Sequence alignment			
**Name of protein**	**Source**	**Percentage Identity with YsaN**	**PDB ID**
YsaN	*Yersinia enterocolitica* subsp. enterocolitica 8081	100 %	NA*
YscN	*Yersinia enterocolitica* subsp. enterocolitica 8081	43.60 %	NA*
Mitochondrial F1-Atpase	Bovine Mitochondrial F1-Atpase chain F	30.85 %	1BMF_F
FliI	flagellum-specific ATP synthase FliI [*Salmonella enterica* subsp. enterica serovar Typhi]	38.28 %	5B0O
Spa47	(plasmid) [*Shigella flexneri*]	42.46 %	5SWJ
EscN	[*Escherichia coli*]	45.26 %	2OBL
HrcN	[*Pseudomonas syringae* pv. *syringae*]	44.38 %	NA*
**(NA*) –** X-Ray structure not available**.**			

Table S 2: List of Proteins used for Multiple Sequence Alignment.

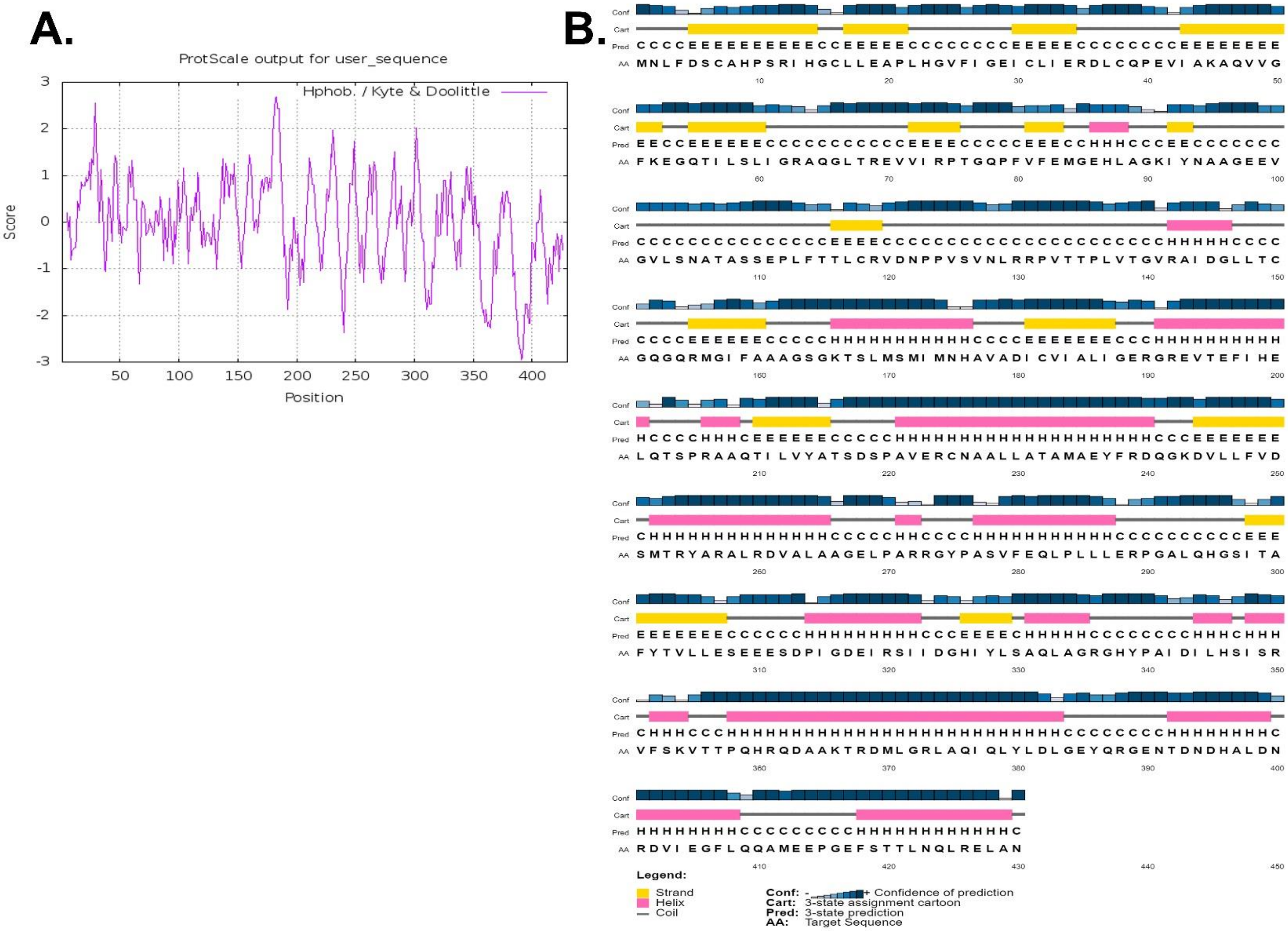

Figure S1: **Bioinformatic analysis of YsaN- A**.- Protscale Kyte & Doolittle hydrophobicity analysis suggesting the N-terminal region as primarily hydrophobic in nature. **B.**- Secondary structure prediction using PSIPRED shows the N-terminal region primarily as a beta-strand.

.

Table S 3: Percent Identity For N-terminal alignment sequence

	YsaN [Y enterocolitica]	YscN [Y enterocolitica]	ChainF [Mitochondrial F1-Atpase]	FliI [S enterica]	Spa47 [Shigella flexneri]	HrcN [Pseudomonas syringae]	EscN [Escherichia coli]
**YsaN [Y enterocolitica]**	**0**						
**YscN [Y enterocolitica]**	21.66	**0**					
**ChainF [Mitochondrial F1-Atpase]**	11.54	12.93	**0**				
**FliI [S enterica]**	16.17	24.24	15.48	**0**			
**Spa47 [Shigella flexneri]**	36.36	22.08	12.24	17.18	**0**		
**HrcN [Pseudomonas syringae]**	16.46	28.85	16.56	23.81	17.09	**0**	
**EscN [Escherichia coli]**	14.88	22.42	15.95	20.69	16.17	18.29	**0**

Table S 4: Percent Identity For C-terminal alignment sequence

	YsaN [Y enterocolitica]	YscN [Y enterocolitica]	ChainF [Mitochondrial F1-Atpase]	FliI [S enterica]	Spa47 [Shigella flexneri]	HrcN [Pseudomonas syringae]	EscN [Escherichia coli]
**YsaN [Y enterocolitica]**	**0**						
**YscN [Y enterocolitica]**	32.10	**0**					
**ChainF [Mitochondrial F1-Atpase]**	13.01	13.82	**0**				
**FliI [S enterica]**	21.69	24.10	12.20	**0**			
**Spa47 [Shigella flexneri]**	30.86	24.69	9.76	24.10	**0**		
**HrcN [Pseudomonas syringae]**	31.76	36.47	15.32	24.71	21.18	**0**	
**EscN [Escherichia coli]**	27.16	38.27	17.07	25.30	27.16	31.76	**0**

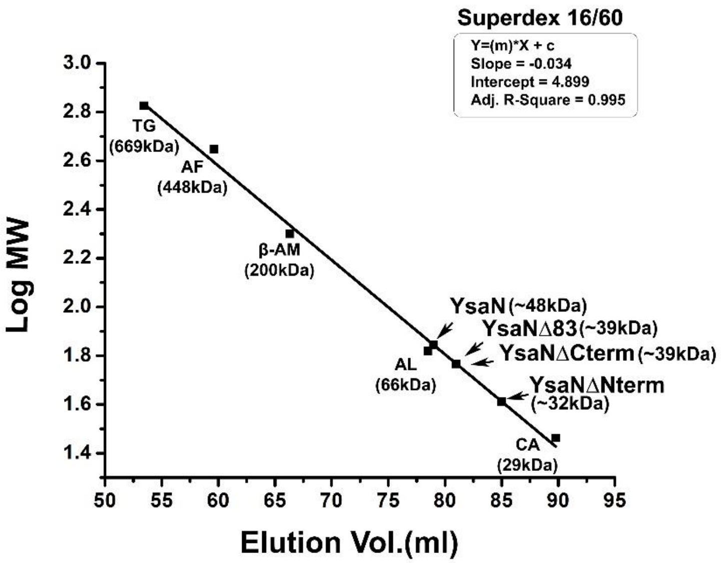

Figure S2: Molecular weight standard curve for Superdex 16/ 60 column: Figure representing elution profiles of all YsaN constructs used in this study on the molecular weight standard curve. Molecular weight standards are represented on the curve as (TG= Thyroglobulin- 669 kDa, AF= Apoferritin 443 kDa, β-AM= β- Amylase- 200 kDa, AL= Albumin- 66 kDa, CA= Carbonic anhydrase- 29 kDa).

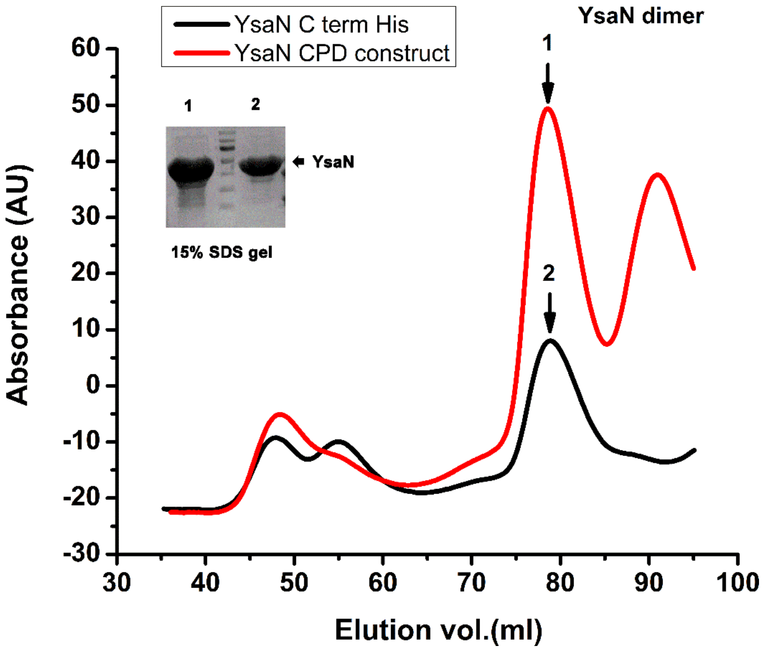

Figures S3: Effect of CPD fusion on the oligomeric state of YsaN- A comparison of C- terminal His tagged YsaN with the YsaN no tag obtained after CPD tag removal. C- terminal CPD fusion of YsaN does not alter the oligomeric status of YsaN in solution.

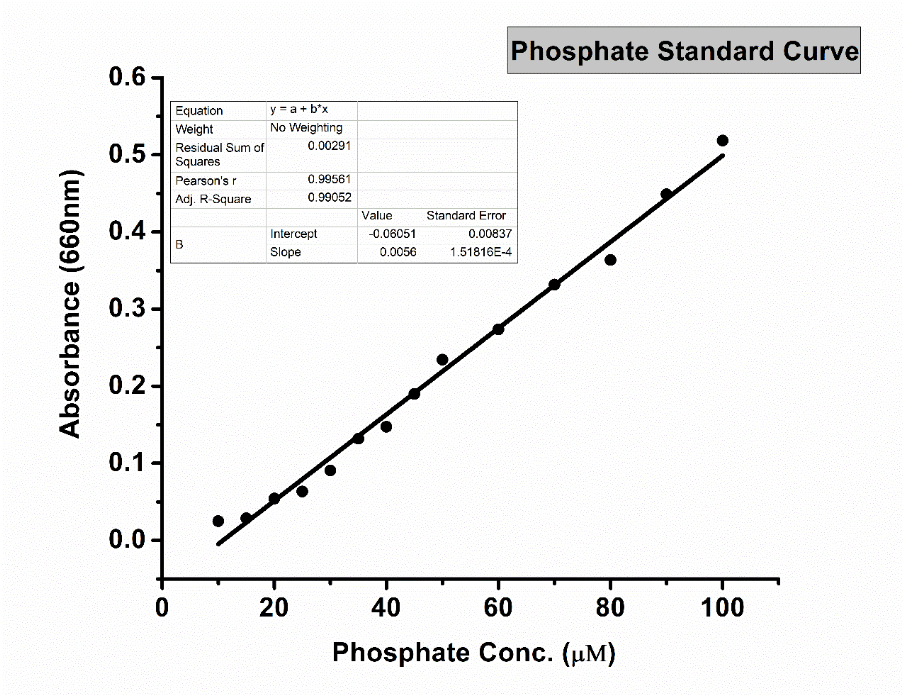

Figures S4: Phosphate standard curve for Malachite green assay- Inorganic phosphate standard curve was calculated from the phosphate standard stock prepared using NaH2PO4 (Sodium phosphate monobasic, Sigma) solution. Absorbance was read at 660nm.

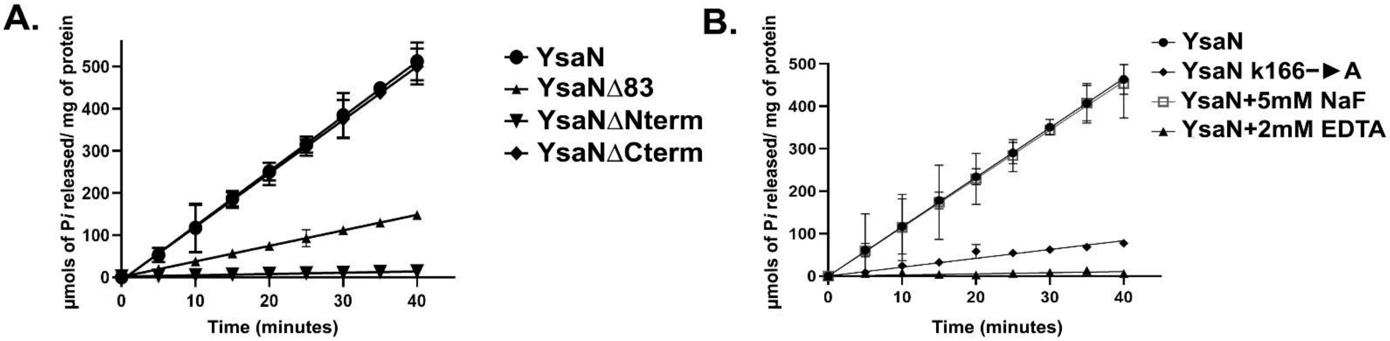

Figure S5: Relative activity assay: A.- Relative activity plot of all YsaN construct used in this study given as μmol of inorganic phosphate (P*i*) released per milligrams of protein. **B**.- Relative activity of YsaN, YsaN K→166A, YsaN in presence of 5mM NaF, and YsaN in presence of 2mM EDTA in the assay buffer.

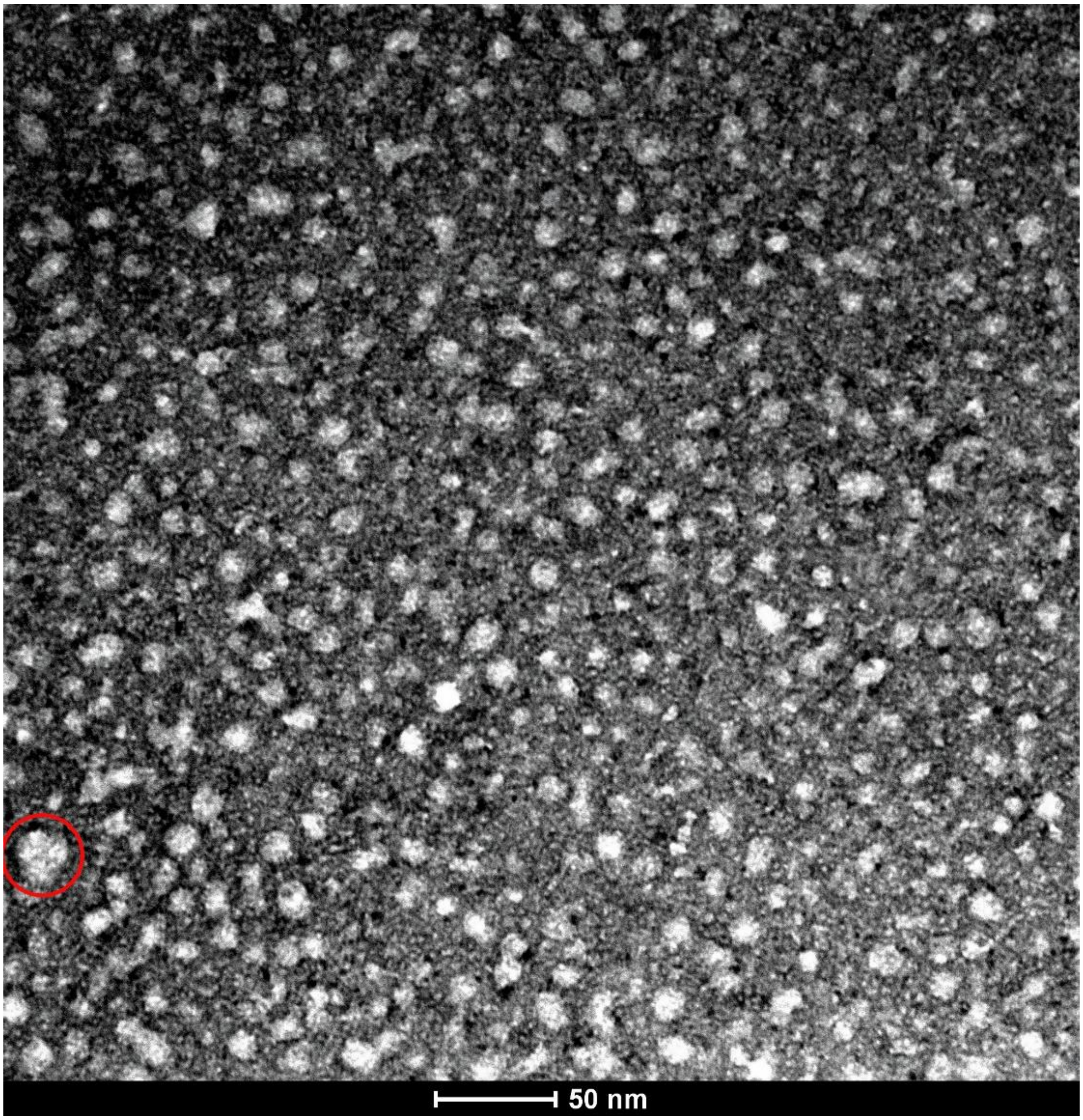

Figure S6: Negative TEM image of YsaN in presence of ADP.AlFX. YsaN oligomers in presence of 1.5mM ADP.AlFX. Many of the oligomers appear as approximately 15-20nm particles. The red circle represents nonspecific aggregates. The prepared sample grids were stained with 1% uranyl acetate.

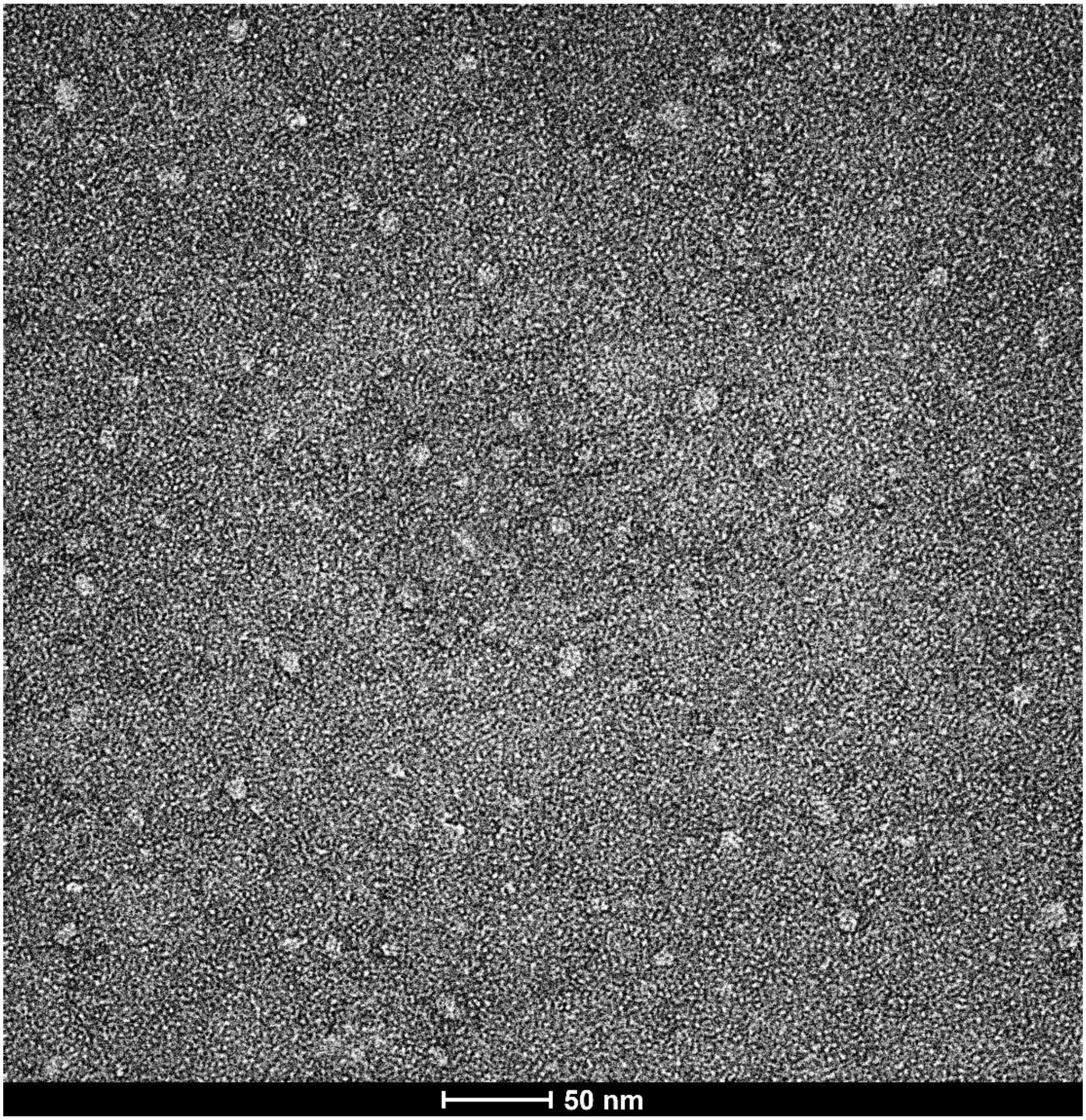

Figure S7: Negative TEM image of YsaNΔ83 oligomer complex with ADP.AlFX- YsaNΔ83 oligomers in presence of 1.5mM ADP.AlFX appears as approximately 10nm particle. The prepared sample grids were stained with 1% uranyl acetate.

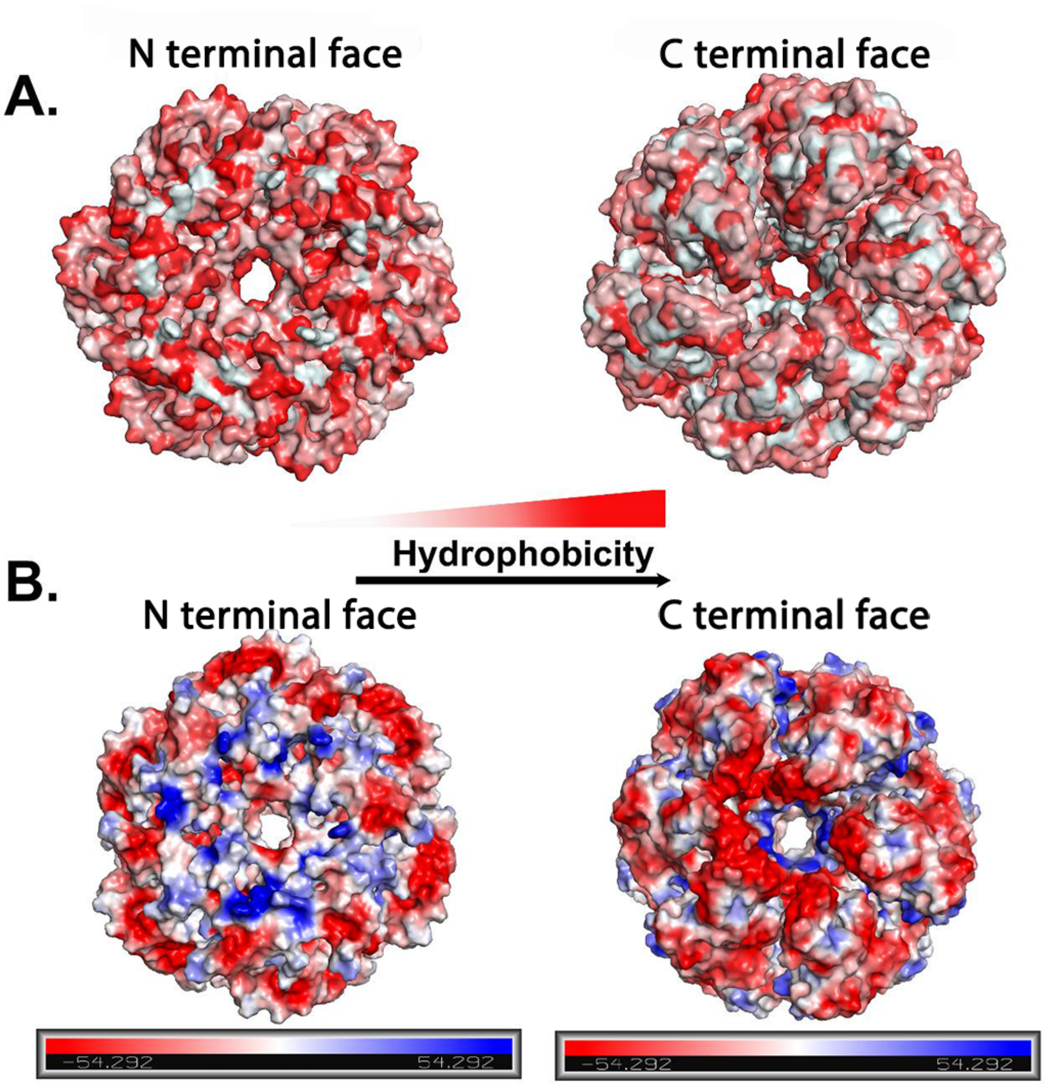

Figures S8: Surface map of YsaN hexameric complex- A.- Hydrophobic surface map of N terminal face and C terminal face respectively of YsaN hexamer complex model (red indicates hydrophobic residue and white indicates hydrophilic residues exposed). **B** - Surface map of N terminal and C terminal face of YsaN hexamer complex based on charge (red indicate negatively charged amino acid residues and blue represents positively charged amino acid residues.

A.

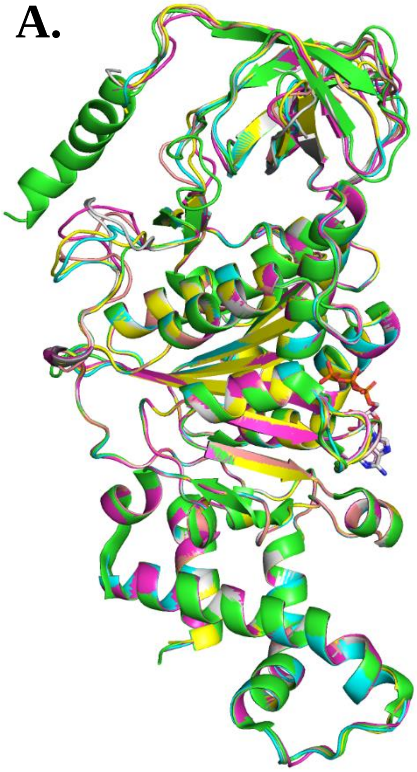

B.

Model Name	DOPE score	RMSD compared to Chain D of FliI-FliH structure (PDB ID-5B0O)
YsaN Model 1	-42719.90625	0.123
YsaN Model 2	-42222.11328	0.128
YsaN Model 3	-43239.66797	0.115
YsaN Model 4	-42660.85938	0.121
YsaN Model 5	-43316.37500	0.122

Table S 5: B. Table represents the variance in the DOPE score of MODELLER generated YsaN models and the RMSD of aligned models with the template FliI. The alignment was done in PyMOL software.

Figure S9: A. Representation of all five models aligned structures generated by MODELLER software with the template FliI. Image generated in PyMOL.

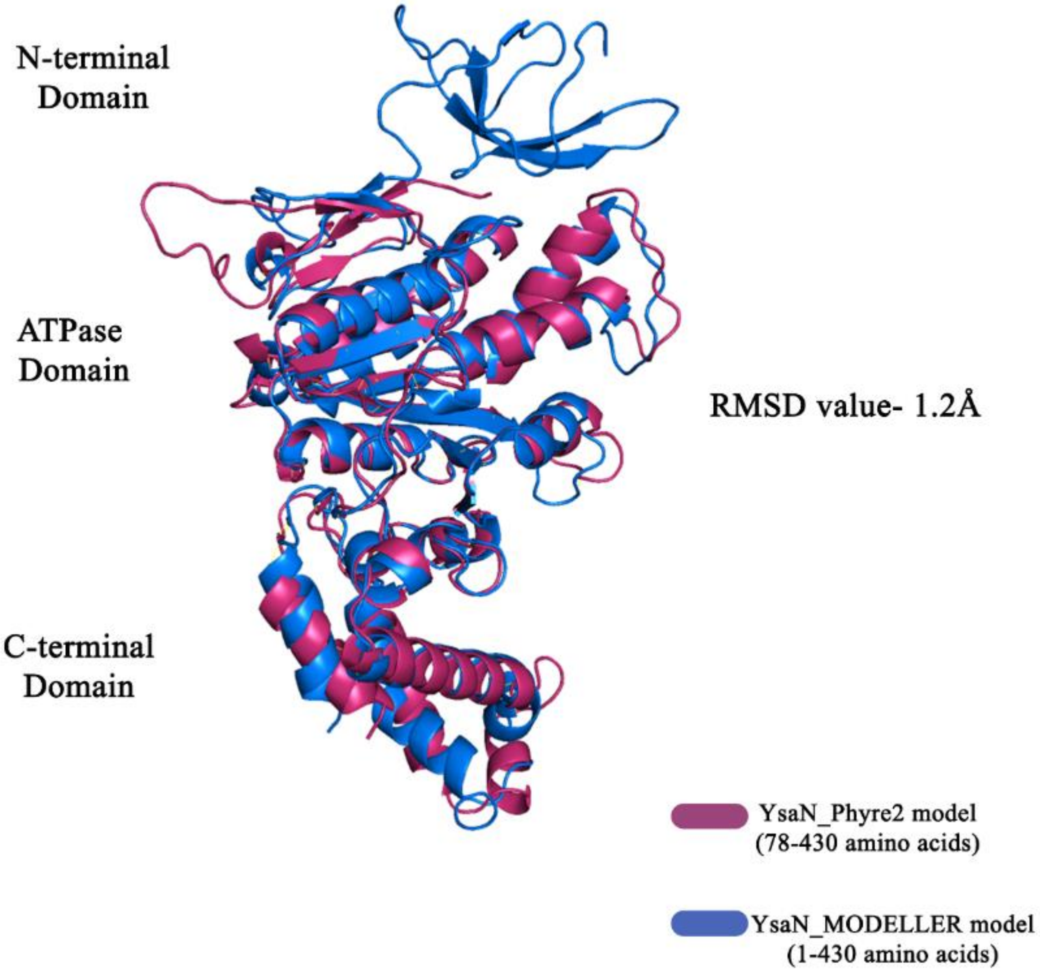

Figure S10: Representation of aligned structures generated by MODELLER software and Phyre2 server. The alignment was done in PyMOL software.

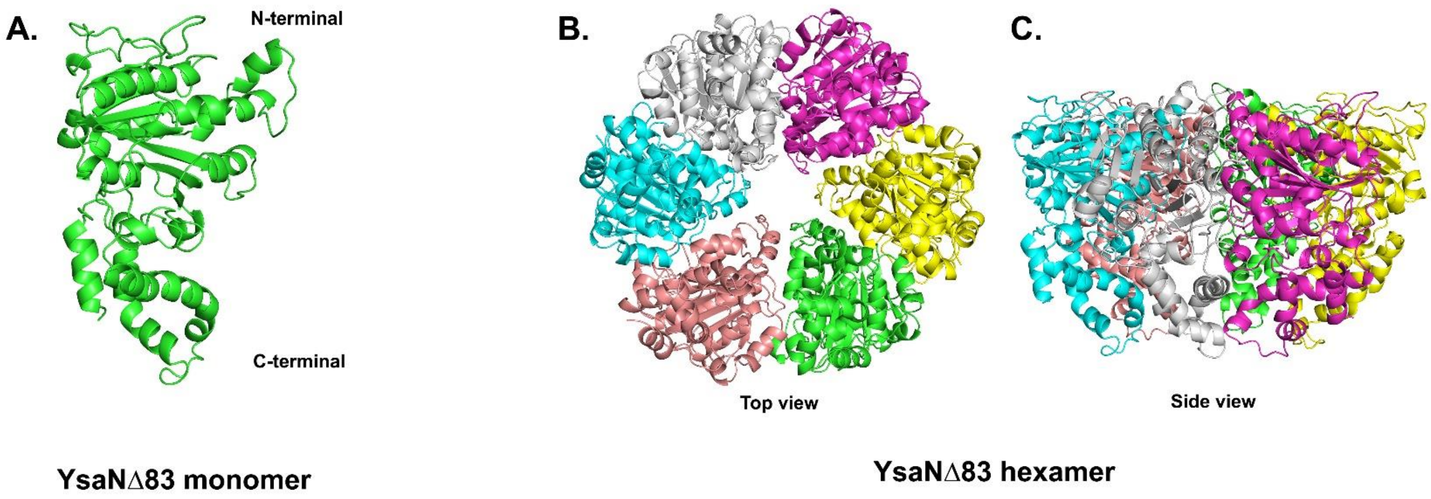

Figure S11: Homology model of YsaNΔ83 monomer and hexamer. A. YsaNΔ83 monomer. **B-C.** YsaNΔ83 hexamer, Top view and Side view respectively.
